# Exercise – induced changes in cerebrospinal fluid miRNAs in Gulf War Illness, Chronic Fatigue Syndrome and sedentary control subjects

**DOI:** 10.1038/s41598-017-15383-9

**Published:** 2017-11-10

**Authors:** James N. Baraniuk, Narayan Shivapurkar

**Affiliations:** Division of Rheumatology, Immunology and Allergy, Department of Medicine, Georgetown University, Washington, District of Columbia United States of America

## Abstract

Gulf War Illness (GWI) and Chronic Fatigue Syndrome (CFS) have similar profiles of pain, fatigue, cognitive dysfunction and exertional exhaustion. Post-exertional malaise suggests exercise alters central nervous system functions. Lumbar punctures were performed in GWI, CFS and control subjects after (i) overnight rest (nonexercise) or (ii) submaximal bicycle exercise. Exercise induced postural tachycardia in one third of GWI subjects (Stress Test Activated Reversible Tachycardia, ***START***). The remainder were Stress Test Originated Phantom Perception (***STOPP***) subjects. MicroRNAs (miRNA) in cerebrospinal fluid were amplified by quantitative PCR. Levels were equivalent between nonexercise GWI (n = 22), CFS (n = 43) and control (n = 22) groups. After exercise, ***START*** (n = 22) had significantly lower miR-22-3p than control (n = 15) and ***STOPP*** (n = 42), but higher miR-9-3p than ***STOPP***. All post-exercise groups had significantly reduced miR-328 and miR-608 compared to nonexercise groups; these may be markers of exercise effects on the brain. Six miRNAs were significantly elevated and 12 diminished in post-exercise ***START***, ***STOPP*** and control compared to nonexercise groups. CFS had 12 diminished miRNAs after exercise. Despite symptom overlap of CFS, GWI and other illnesses in their differential diagnosis, exercise-induced miRNA patterns in cerebrospinal fluid indicated distinct mechanisms for post-exertional malaise in CFS and ***START*** and ***STOPP*** phenotypes of GWI.

## Introduction

Chronic Fatigue Syndrome (CFS)^1–4^ and Gulf War Illness (GWI)^5–8^ are nociceptive, interoceptive, fatiguing illnesses that are currently defined by symptoms and exclusion of other conditions in their extensive differential diagnoses^9^. CFS developed from medical traditions of neurasthenia^10^ and viral infection^11^, and GWI from “signs, symptoms, and ill-defined conditions (SSID; International Classification of Diseases-9th Revision, Clinical Modification (ICD-9-CM) codes 780-799)”^12^. These legacies are being revised based on new discoveries about disease pathogenesis^13–18^. With the revisions comes an increasing need for objective biomarkers to define and diagnose these diseases.

**Figure 1:**
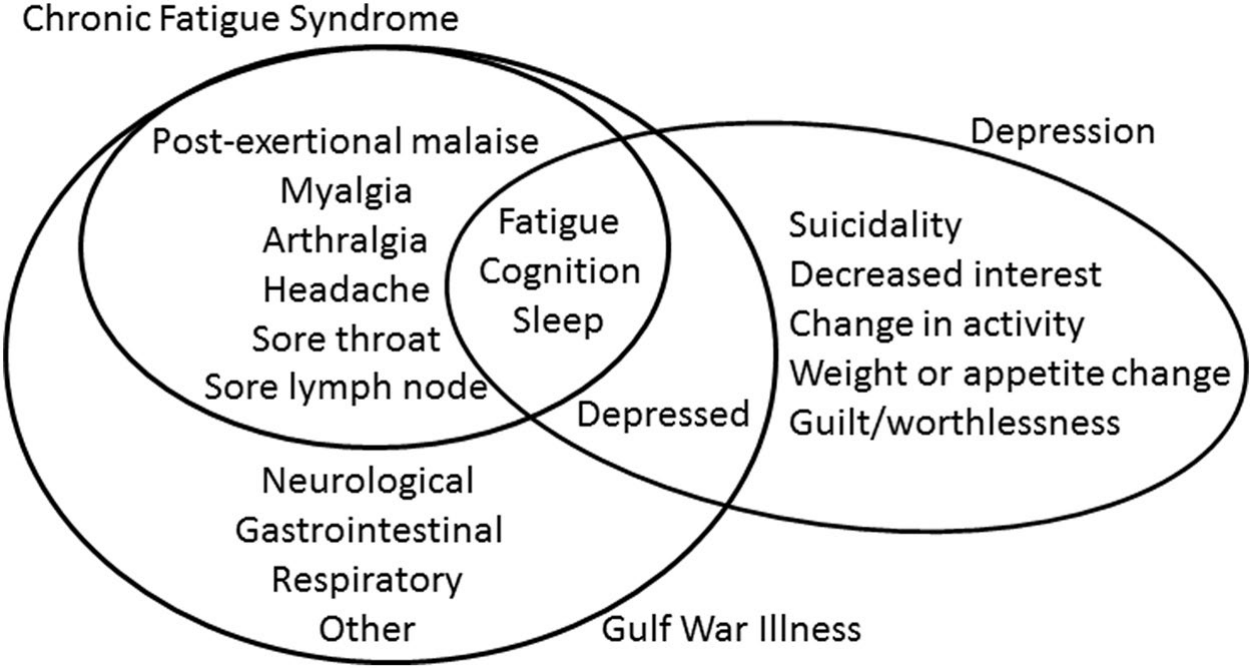
Overlap of diagnostic criteria for CFS^1^, GWI^7^ and major depressive disorder^78^. Diagnostic protocols for CFS, GWI and depression selected different sets of primary and ancillary symptoms. CFS requires fatigue, then confirmation with ≥4 of 8 ancillary criteria. Active depression and other psychiatric diseases are exclusionary diagnoses for CFS. GWI requires 3 of 7 categories of symptoms. Depression requires depressed affect and anhedonia, then sufficient supporting complaints.

The 1994 Center for Disease Control (CDC) criteria for CFS are: (a) moderate or severe, persistent and sustained fatigue lasting more than 6 months and causing impairment of daily activities, plus (b) moderate or severe complaints of at least 4 of 8 ancillary criteria: short term memory or problems with concentration, sore throat, sore lymph nodes, myalgia, arthralgia, sleep disturbances, new onset headaches that include migraine, and post-exertional malaise (Fig. 1)^1^. Post-exertional malaise, also referred to as exertional exhaustion, is an unique characteristic of CFS^1–4^ that is shared by GWI subjects^7^.

Twenty six years after the First Persian Gulf War, 25% to 32% of the nearly 697,000 U.S. veterans of that conflict continue to have cognitive and physical exhaustion that is made worse by effort (exertional exhaustion), systemic pain and hyperalgesia, migraines, gastrointestinal distress with severe diarrhea, and other medical problems (Table 1)^7,8^. Epidemiological risk factors include exposures to low dose sarin and cyclosarin from munitions such as the demolition at Khamisiyah, Iraq; pyridostigmine bromide taken for nerve agent prophylaxis; and personal pesticides^19^. Objective findings that distinguish GWI subjects from their unaffected deployed and nondeployed peers and civilians include cerebrospinal fluid proteomics^20^, low activity butyrylcholinesterase alleles in GWI cases who used pyridostigmine (odds ratio = 40.0)^21^, brain white matter dysfunction with increased axial diffusivity by diffusion tensor imaging^22,23^, and mitochondrial dysfunction^18,24^. A logical hypothesis is that Gulf War era exposures to cholinesterase inhibitors^19^ caused acute acetylcholine neurotoxicity in persons with genetically reduced levels of acetylcholinesterase activity^21^, followed by chronic progression of the initial lesions^18,20,22–24^.

**Table 1:** Demographics and symptom severities. Mean ± SD.

Group	Non-exercise groups			Post-exercise groups			
	*sc0*	*cfs0*	*gwi0*	*SC*	*START*	*STOPP*	*CFS*
N	22	43	22	15	22	42	16
Age	42.0 ± 13.1	45.5 ± 10.6	49.2 ± 9.7	44.3 ± 11.5	45.3 ± 8.7	46.6 ± 8.2	47.0 ± 10.6
Male*	11 (48%)	9 (22%)	9 (41%)	11 (85%)	8 (80%)	18 (82%)	2 (29%)
CFS Severity Questionnaire (CFSQ)							
Fatigue	1.3 ± 1.4	3.7 ± 0.4^†^	3.5 ± 0.5^‡^	1.2 ± 0.9	3.7 ± 0.5^§^	3.5 ± 0.5^¶^	3.9 ± 0.4^#^
memory, concentration	1.0 ± 1.2	3.1 ± 0.7^†^	2.8 ± 1.0^‡^	1.0 ± 1.2	3.1 ± 0.9^§^	2.9 ± 0.8^¶^	2.7 ± 1.0^#^
sore throat	0.3 ± 0.8	1.5 ± 1.2^†^	1.5 ± 1.1^‡^	0.2 ± 0.6	2.1 ± 1.1^§^	1.4 ± 1.3	1.7 ± 1.3^#^
sore lymph nodes	0.2 ± 0.5	1.4 ± 1.3^†^	1.8 ± 1.5^‡^	0.1 ± 0.3	1.9 ± 1.3^§^	1.0 ± 1.1	1.3 ± 1.4
muscle pain	1.3 ± 1.4	3.1 ± 1.1^†^	3.0 ± 1.3^‡^	0.9 ± 1.0	3.3 ± 0.5^§^	3.1 ± 0.9^¶^	2.9 ± 1.2^#^
joint pain	0.9 ± 1.1	2.6 ± 1.3^†^	2.8 ± 1.2^‡^	1.1 ± 1.0	3.2 ± 1.3^§^	3.2 ± 0.8^¶^	2.6 ± 1.1
headache	1.0 ± 1.4	2.5 ± 1.3^†^	2.5 ± 1.2^‡^	1.0 ± 1.4	3.3 ± 1.1^§^	2.4 ± 1.2^¶^	1.7 ± 1.7
disrupted sleep	1.4 ± 1.5	3.5 ± 0.7^†^	3.6 ± 0.6^‡^	1.5 ± 1.2	3.6 ± 0.5^§^	3.6 ± 1.0	3.3 ± 0.5^#^
exertional exhaustion	1.3 ± 1.6	3.3 ± 1.0^†^	3.4 ± 1.2^‡^	0.4 ± 0.8	3.7 ± 0.5^§^	3.3 ± 0.8^¶^	3.4 ± 0.5^#^
CFSQ Sum8	7.4 ± 7.0	21.1 ± 4.6^†^	21.4 ± 5.2^‡^	6.2 ± 5.6	24.2 ± 3.7^§^	20.9 ± 5.1^¶^	19.6 ± 5.6^#^
SF-36 Quality of Life							
physical function	83.0 ± 26.0	38.0 ± 20.5^†^	44.0 ± 26.9^‡^	87.3 ± 22.9	43.3 ± 27.2^§^	43.1 ± 23.2^¶^	36.4 ± 19.7^#^
role physical	59.8 ± 47.5	6.4 ± 19.5^†^	8.8 ± 18.6^‡^	86.5 ± 30.0	0.0 ± 0.0^§^	15.5 ± 29.0^¶^	0.0 ± 0.0^#^
bodily pain	73.0 ± 32.7	31.0 ± 20.0^†^	38.0 ± 26.1^‡^	75.3 ± 21.5	20.1 ± 15.6^§^	30.5 ± 18.4^¶^	39.0 ± 25.7^#^
general health	67.5 ± 20.6	32.7 ± 19.0^†^	31.8 ± 19.7^‡^	75.5 ± 13.0	17.4 ± 12.7^§^	29.4 ± 19.8^¶^	24.3 ± 14.3^#^
vitality	54.6 ± 28.6	11.6 ± 11.7^†^	20.5 ± 13.5^‡^	63.8 ± 12.4	12.2 ± 11.8^§^	14.0 ± 13.6^¶^	8.6 ± 9.4^#^
social function	79.9 ± 29.6	24.6 ± 21.1^†^	28.1 ± 21.8^‡^	84.6 ± 15.4	18.1 ± 16.7^§^	30.4 ± 24.5^¶^	19.6 ± 12.2^#^
Chronic Multisymptom Index (CMSI)							
rheumatic	7.0 ± 9.2	19.2 ± 6.9^†^	21.3 ± 9.6^‡^	3.9 ± 4.2	23.1 ± 7.2^§^	20.9 ± 5.1^¶^	20.3 ± 7.5^#^
dyspnea	2.0 ± 3.4	6.8 ± 6.3^†^	8.2 ± 5.2^‡^	1.7 ± 2.0	12.0 ± 6.1^§^	7.0 ± 4.5^¶^	9.1 ± 5.2^#^
neurological	2.0 ± 3.7	7.5 ± 3.8^†^	8.3 ± 4.0^‡^	1.1 ± 1.3	9.2 ± 3.4^§^	8.7 ± 2.5^¶^	7.4 ± 3.5^#^
CMSI Sum52	24.2 ± 28.9	58.4 ± 25.3^†^	77.2 ± 34.1^‡^	12.6 ± 11.2	89.7 ± 26.1^§^	72.2 ± 23.5^¶^	64.3 ± 30.4^#^
CESD	9.2 ± 8.1	20.9 ± 9.0^†^	27.7 ± 12.9^‡^	8.8 ± 7.6	33.2 ± 8.4^§^	24.0 ± 10.8^¶^	17.9 ± 10.0^#^
%CESD ≥ 16	19.0%	68.6%	81.0%	33.3%	85.7%	73.2%	53.3%
GAD7	0.50 ± 0.02	0.17 ± 5.54	8.83 ± 6.77^‡^	4.17 ± 6.00	2.25 ± 4.77^§^	7.81 ± 5.81	4.67 ± 5.39
FM 1990*	0/22 (5%)	15/38 (39%)	9/19 (47%)	0/13 (0%)	5/10 (50%)	9/22 (41%)	2/6 (33%)
IgG/albumin	0.13 ± 0.04	0.11 ± 0.03	0.12 ± 0.03	0.12 ± 0.02	0.13 ± 0.04	0.12 ± 0.03	0.14 ± 0.03

*p < 0.002 by contingency tables between all groups.

FDR < 0.010 after significant ANOVA: ^†^sc0 vs cfs0; ^‡^sc0 vs gwi0; ^§^SC vs START; ^¶^SC vs STOPP; ^#^START vs CFS.

We have reported that GWI veterans can be divided into two phenotypes based on responses to the physiological stressor of submaximal exercise testing^25^. One third of subjects developed new postural tachycardia after exercise and were positively selected as the ***START*** (Stress Test Activated Reversible Tachycardia) group. ***START*** subjects had: (i) exercise - induced postural tachycardia, (ii) increased blood oxygenation level dependent (BOLD) signal in the cerebellar vermis during a cognitive task before exercise, (iii) reduced BOLD signals during a working memory task after exercise, and (iv) reduced brainstem volumes suggesting atrophy. The remainder formed the Stress Test Originated Phantom Perception (***STOPP***) group because they had significantly greater BOLD activation of basal ganglia and anterior insula during cognitive testing than sedentary controls (SC) and ***START ***^25^. That pattern was similar to phantom limb pain^26^. The two phenotypes suggest there were two mechanisms of initial injury or on-going progression that will require different diagnostic and treatment approaches.

Cerebrospinal fluid was extensively assayed for micro-RNAs (miRNA), proteomics^20^, metabolomics, and other analytes to interrogate the central neurotoxic pathologies proposed in GWI^27^ and CFS^16,17^. miRNAs are ~22 nucleotide long, single-stranded RNAs transcribed from genomic DNA^28^. They form the RNA-induced silencing complex (RISC) and bind to complementary sequences in the 3′ untranslated region of mRNAs to repress translation or promote mRNA degradation. miRNAs dynamically fine-tune the expression of most cellular proteins. Quantitative polymerase chain reaction (QPCR) was used to measure cerebrospinal fluid miRNAs.

First, we hypothesized that CFS (***cfs0***), GWI (***gwi0***) and sedentary control (***sc0***) subjects at rest (nonexercise, lower case italics with 0) would have significant differences in cerebrospinal fluid biomarkers from each other. The nonexercise groups rested overnight and had no exercise before their lumbar punctures. Nonexercise miRNA patterns were predicted to be different from other conditions in the differential diagnosis such as depression and fibromyalgia. Second, differences would be magnified in post-exercise ***SC***, ***CFS***, and the exercise-defined ***START*** and ***STOPP*** phenotypes of GWI subjects when compared to each other (upper case italics to denote post-exercise). Third, differences between the post-exercise groups and their appropriate nonexercise comparison groups (***SC*** vs. ***sc0***, ***CFS*** vs. ***cfs0***, ***START*** vs. ***gwi0***, ***STOPP*** vs. ***gwi0***) would model the effects of exercise on the central nervous system and the pathology of exertional exhaustion.

## Methods

### Clinical information

All subjects gave written informed consent. The protocol was approved by the Georgetown University Institutional Review Board and the Human Research Protection Office of the Department of Defense Congressionally Directed Medical Research Program. Lumbar puncture, quantitative PCR, and other investigations were performed in accordance with currently published standards, guidelines (MIQE Guidelines: Minimum Information for Publication of Quantitative Real-Time PCR Experiments http://miqe.gene-quantification.info/) and World Medical Association Declaration of Helsinki - Ethical Principles for Medical Research Involving Human Subjects (http://www.wma.net/en/30publications/10policies/b3/). The investigations were not considered clinical trials using the World Health Organization (WHO) definition (http://www.who.int/ictrp/en/).

**Figure 2:**
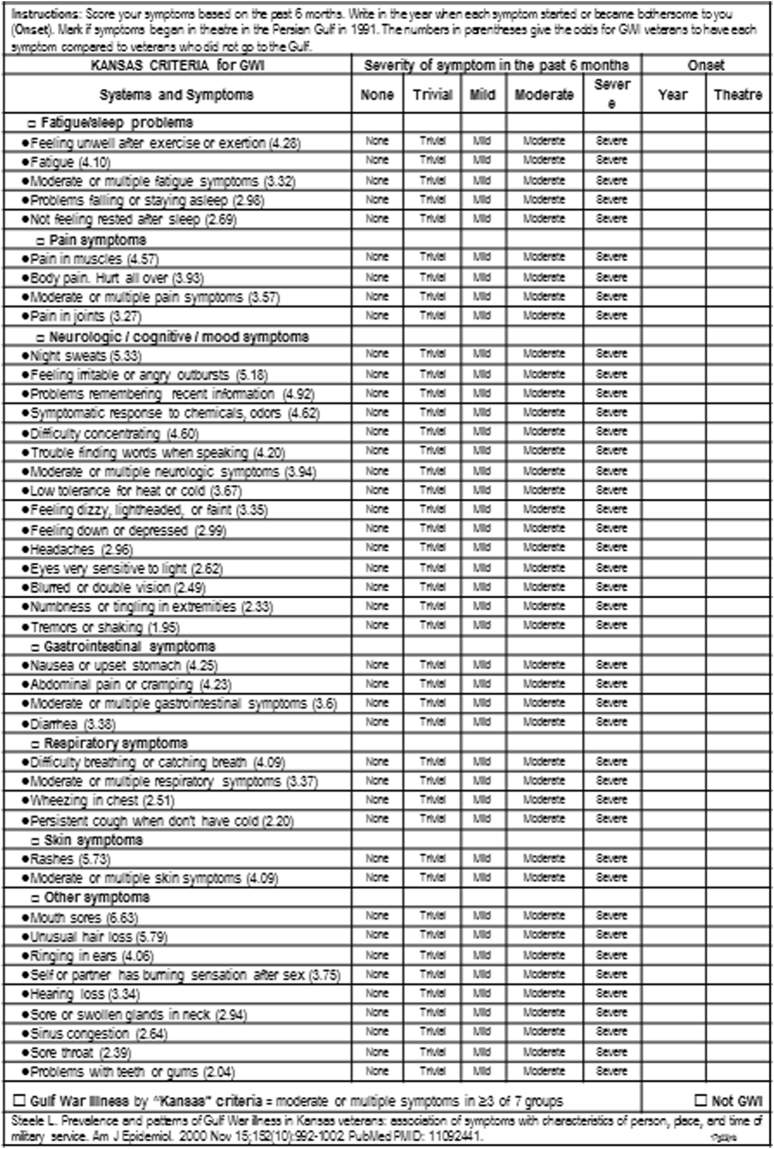
Kansas Criteria for Gulf War Illness scoring form based on Steele^7^. ©*JNBaraniukMD_17g13*. *Used with permission of the copyright holder*.

**Figure 3:**
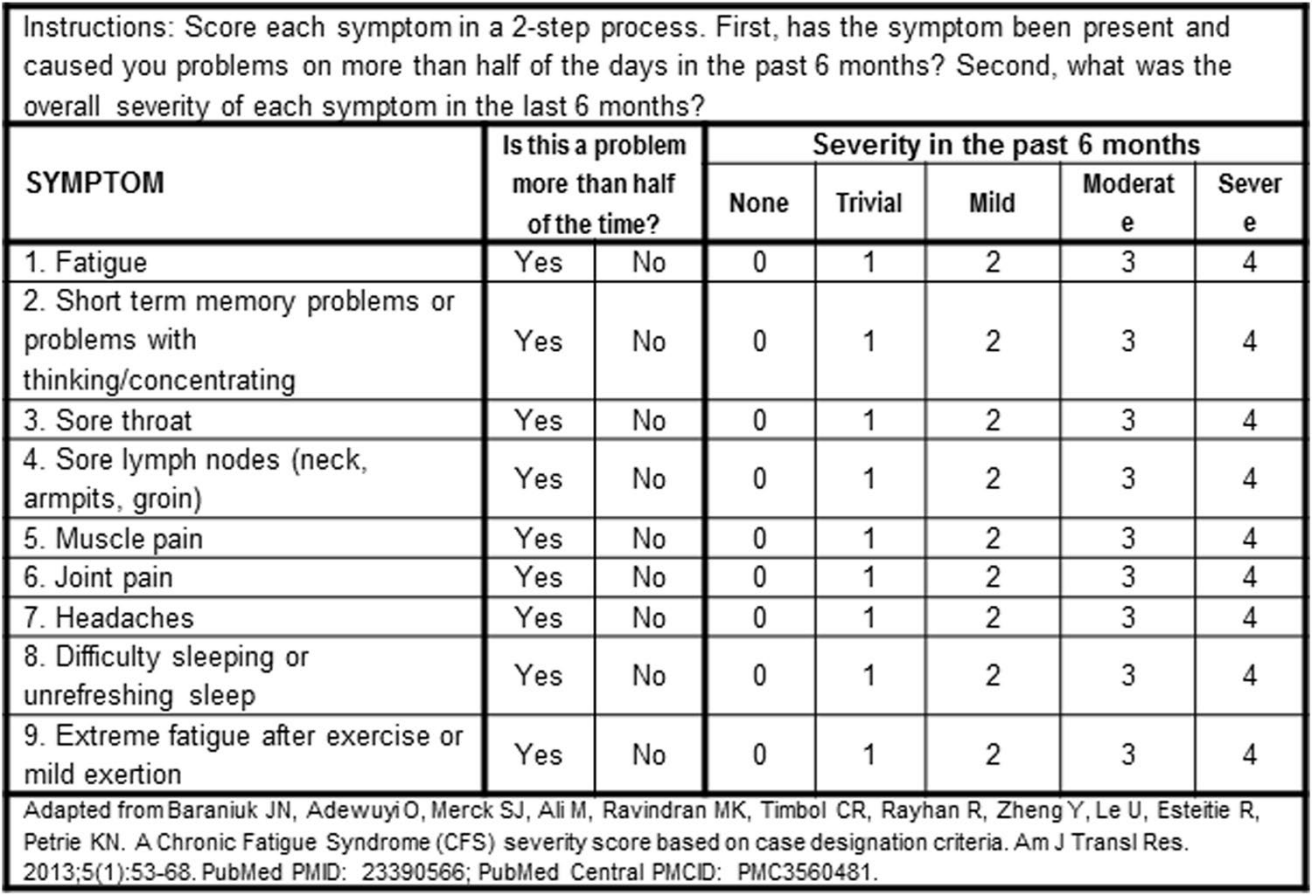
Chronic Fatigue Syndrome Symptom Severity Questionnaire. ©*JNBaraniukMD_17g13 Used with permission of the copyright holder*.

GWI and healthy veterans, and healthy, non-military, sedentary control (***SC***) subjects completed questionnaires for case designation criteria of GWI (Fig. 2)^7^ and CFS (Fig. 3)^1,29^, common symptoms in CFS and GWI^30^, quality of life^31^, Generalized Anxiety Disorder 7^32^, and Center for Epidemiologic Studies Depression Scale^33^ scores. Fibromyalgia was assessed by pain plus tenderness (1990 criteria)^34^. Clinical and methodological details were published previously^25^. All subjects had submaximal bicycle exercise stress tests on 2 consecutive days with magnetic resonance imaging before and afterwards, followed by a lumbar puncture^25^. Subjects cycled at 70% of age predicted maximum heart rate for 25 min^35^ followed by stepwise increases in bicycle resistance to reach 85% predicted heart rate^25^. Exercise was required to induce postural tachycardia that defined the ***START*** phenotype. Cerebrospinal fluid total protein, albumin and IgG were measured^36^ and aliquots were frozen at −80 °C until thawed for miRNA extraction.

### Quantitative PCR

miRNA analysis was completed in blinded fashion by N.S. without knowledge of subject diagnosis. Total RNA was isolated by mixing 0.5 ml cerebrospinal fluid 1:10 with QIAzol™ lysis reagent (Qiagen) and 0.1 ml CHCl_3_ before vortexing for 1 min^37–39^. miRNA was extracted from the upper phase using miRNAeasy Mini Kits (Qiagen). miRNA levels were estimated by optical density of cDNA after reverse transcription with miScriptII RT kits (Qiagen). miRNA expression profiling used miScript PCR arrays for 380 miRNAs and miScript SYBRgreen PCR kits (Qiagen) on an ABI 7900 HT Real-Time PCR system (Applied Biosystems) and manufacturer’s protocol.

### miRNA selection

The first level of constraint required miRNAs to be detectable with PCR cycle threshold (Ct) ≤ 35. miRNAs with Ct > 35 were designated as “undetectable” and 35 was imputed as their Ct. Second, in order to be considered a viable biomarker, miRNA had to detectable with Ct ≤ 35 in at least two thirds of subjects in a group.

### miRNA normalization and ΔΔCt

Four normalization strategies and ΔΔCt computations were compared.

The N0 (no normalizer) analysis used the entire dataset with 35 imputed for all Ct > 35. The average ΔCt was calculated for each group, then ΔΔCt determined between each pair of groups^40^. Significantly different miRNAs were detected by one-way ANOVA followed by Tukey’s Honest Significant Difference (HSD; p < 0.05). Student’s 2-tailed unpaired t-tests were computed for all miRNAs and pairs of groups, and False Discovery Rates (FDR) calculated to correct for multiple comparisons^41^. FDR ≤ 0.10 was used as the next constraint to detect significant differences in ΔΔCt. The other normalizers used 2 (N2), 3 (N3) and 6 (N6) miRNAs. For each individual, ΔCt was calculated as the difference of the N2, N3 or N6 normalizer minus the Ct for each of the other miRNAs. Average ΔCt and ΔΔCt were calculated.

Outcomes were: (i) differences between non-exercise groups (***sc0***, ***cfs0*** and ***gwi0***), (ii) differences between groups after exercise (***SC***, ***START***, ***STOPP***, ***CFS***), and (iii) exercise–induced differences between each post-exercise group and its appropriate non-exercise control group. ΔΔCt data were reported as mean ± SD to allow calculation of Cohen’s d (mean difference/SD_pooled_) and to predict future sample sizes^42^. Receiver operating characteristics were calculated for each significant ΔΔCt in SPSS 24.

## Results

### Demographics

CFS groups (***cfs0***, ***CFS***) had more females, and GWI groups (***gwi0***, ***START***, ***STOPP***) more males (Table 2). Quality of life^31^, fatigue, cognitive, sleep, pain and interoceptive symptoms^29,30^ were significantly impaired in GWI and CFS groups compared to sedentary control subjects. Fibromyalgia (1990 criteria) was more prevalent in CFS and GWI than controls^34^. The case designation criteria of GWI, CFS, generalized anxiety and depression share fatigue, sleep, cognition, and sympathetic nervous system symptoms^32^. The Center for Epidemiology – Depression questionnaire found depression in 78.3% of GWI, 64.0% of CFS and 25.0% of control subjects^33^. Generalized Anxiety Disorder 7 scores were significantly higher for ***gwi0*** and ***START*** compared to ***sc0*** (FDR < 0.01) and ***SC*** (FDR < 0.01), respectively^33^. Cerebrospinal fluid total protein, albumin, IgG and their ratios were equivalent between groups^36^.

**Table 2:** Receiver operating characteristics.

	low Ct (high miRNA)	High Ct (low miRNA)	Specificity	Threshold	Sensitivity	AUC
miR-22-3p	SC	sc0	0.80	32	0.80	0.81
miR-204-5p	SC	sc0	0.80	31	0.80	0.91
miR-99b-5p	SC	sc0	0.73	33	0.73	0.82
miR-30d-5p	SC	sc0	0.72	33	0.72	0.89
miR-425-3p	SC	sc0	0.7	33	0.7	0.79
miR-328	sc0	SC	0.74	23	0.74	0.79
miR-608	sc0	SC	0.78	28	0.78	0.80
miR-99b-5p	START	gwi0	0.76	33	0.76	0.84
miR-425-3p	START	gwi0	0.72	33	0.72	0.84
miR-370	START	gwi0	0.72	31	0.72	0.84
miR-328	gwi0	START	0.91	25	0.91	0.98
let-7i-5p	gwi0	START	0.78	33	0.78	0.80
miR-200a-5p	gwi0	START	0.83	33	0.83	0.88
miR-608	gwi0	START	0.82	30	0.82	0.88
miR-93-3p	gwi0	START	0.77	34	0.77	0.84
miR-204-5p	STOPP	gwi0	0.62	31	0.62	0.72
miR-99b-5p	STOPP	gwi0	0.75	33	0.75	0.84
miR-328	gwi0	STOPP	0.86	23	0.86	0.87
let-7i-5p	gwi0	STOPP	0.72	32	0.72	0.78
miR-200a-5p	gwi0	STOPP	0.71	31	0.71	0.73
miR-608	gwi0	STOPP	0.79	28	0.79	0.77
miR-93-3p	gwi0	STOPP	0.75	32	0.75	0.76
miR-328	cfs0	CFS	0.84	24	0.84	0.90
miR-608	cfs0	CFS	0.76	29	0.76	0.84
let-7i-5p	cfs0	CFS	0.68	31	0.68	0.74
miR-200a-5p	cfs0	CFS	0.72	32	0.72	0.82
miR-93-3p	cfs0	CFS	0.77	33	0.77	0.83
miR-126-5p	cfs0	CFS	0.74	32	0.75	0.88
miR-19b-3p	cfs0	CFS	0.63	34	0.63	0.79
miR-505-3p	cfs0	CFS	0.65	34	0.65	0.82
miR-92a-3p	cfs0	CFS	0.81	29	0.81	0.86
miR-186-3p	cfs0	CFS	0.67	35	0.67	0.79
miR-323b-5p	cfs0	CFS	0.75	32	0.75	0.84
miR-532-5p	cfs0	CFS	0.70	34	0.70	0.73

### Normalizers

The raw data were placed in Supplementary Table S1. The statistical constraints reduced the number of miRNAs that were candidates for biomarkers and normalizers down to 88 (Supplementary Table S2).

Supplementary Tableas the raw Ct data with 35 imputed for all Ct > 35 (Supplementary Table S3).

**Figure 4:**
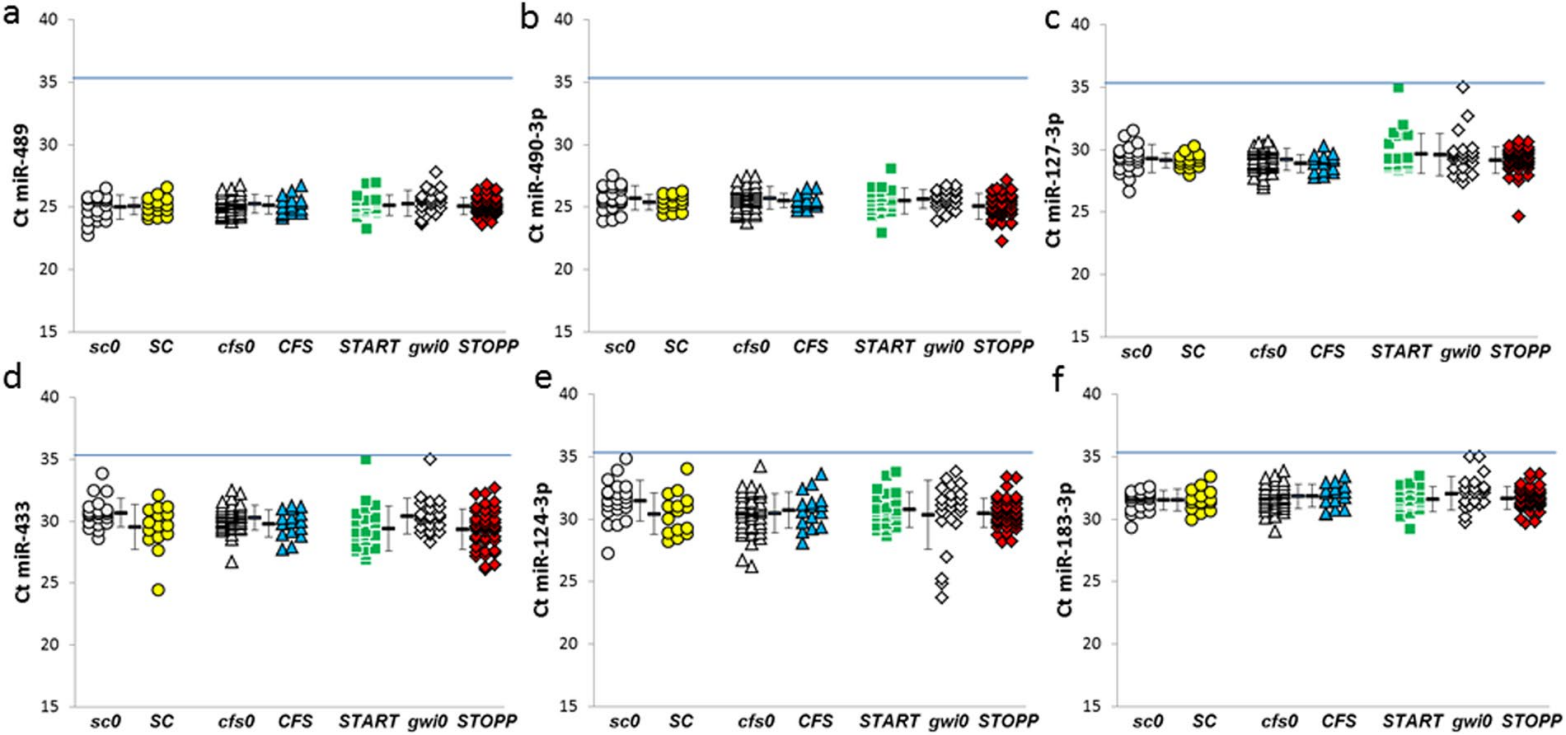
Normalizer miRNAs. Data are shown as Ct for each miRNA (N0 normalizer). The N2 normalizer used (**a**) miR-489 and (**b**) miR-490-3p. N3 added (**c**) miR-127-3p. The N6 normalizer added (**d**) miR-433, (e) miR-124-3p, and (**f**) miR-183-3p. Each miRNA had ΔΔCt < 1.0 between groups, ANOVA > 0.05, FDR > 0.10, and were detectable in at least 180 of the 182 subjects. The blue line indicates Ct = 35. Nonexercise groups were control (***sc0***, grey circles), Chronic Fatigue Syndrome (***cfs0***, grey triangles), and Gulf War Illness (***gwi0***, grey diamonds). Post-exercise groups were control (***SC***, yellow circles), Chronic Fatigue Syndrome (***CFS***, blue triangles), and the Gulf War Illness ***START*** (green squares) and ***STOPP*** (red diamonds) phenotypes. Mean ± SD.

The N2 normalizer used miR-489 and miR-490-3p because they (i) were detected in all subjects with Ct ≤ 35, (ii) were abundant in cerebrospinal fluid, (iii) had small variances (25.2 ± 0.8 and 25.5 ± 0.9, respectively, mean ± SD) with narrow ranges for Ct (minimum 22.8 to maximum 27.8, and minimum 22.3 to maximum 28.1, respectively), and (iv) were not significantly different between groups (ANOVA > 0.05 and FDR > 0.10 for each pairing) (Fig. 4).

The N3 normalizer was the mean of miR-489, miR-490-3p and miR-127-3p (29.3 ± 1.2, mean ± SD, minimum 24.2, maximum 35). Two Ct values were 35; the averages for the 2 groups were imputed instead of 35 for this normalizer (26.6 ± 2.1, mean ± SD).

The N6 normalizer added miR-124-3p (30.6 ± 1.7, mean ± SD), miR-183-3p (31.8 ± 1.0, mean ± SD), and miR-433 (29.9 ± 1.5, mean ± SD). Two subjects each had 35 imputed for miR-127-3p, miR-183-3p and miR-433; the N6 normalizer included these values (28.7 ± 2.8, mean ± SD). Ct data for these 6 miRNAs were shown in Fig. 4.

All normalizer miRNAs had ΔΔCt < 1.0, ANOVA > 0.05, FDR > 0.10 and were detectable in at least 180 of the 182 subjects (Supplementary Table S2).

**Figure 5:**
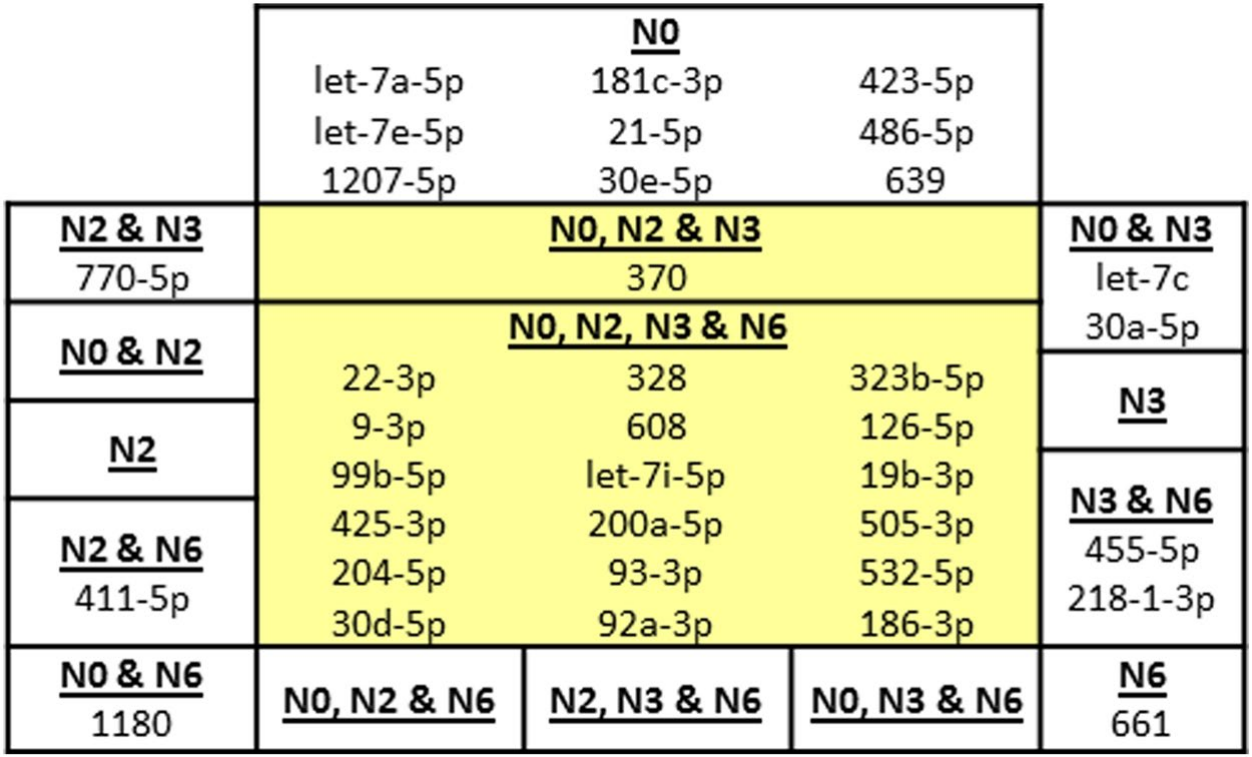
Intersection of miRNAs from each normalizer. The intersection of N0, N2, N3, and N6 identified 16 miRNAs that had at least 1 significant difference between groups (central yellow boxes). N0, N2 and N6 and N2, N3 and N6 added one each. Pairs of normalizers identified 6 miRNAs that were not considered significant. N0 was least selective as it identified an additional 12 miRNAs that were considered false positives. Therefore, the miRNAs selected by 3 or 4 normalizers were the set of significantly different miRNAs.

The N0 normalizer selected 31 miRNAs that met the significance criteria of (i) being detected with Ct ≤ 35 in more than two thirds of subjects in at least 1 of the 7 groups, (ii) HSD ≤ 0.05, and (iii) FDR ≤ 0.10. N2 identified 21, N3 had 24, and N6 found 23 significant miRNAs. The intersection of the 4 normalizers identified 18 miRNAs with at least 1 significant difference between groups (Fig. 5). One was added by the intersection of 3 normalizers. There was excellent agreement for the magnitudes of ΔΔCt between the 4 normalizers (Supplementary Tables S4 and S5). In contrast, N0 selected 9 additional miRNAs that were not found with N2, N3 and N6 (Fig. 5). These were considered false positive results.

### Nonexercise groups

None of the miRNAs were significantly different between nonexercise groups using our stringent criteria. miR-22-3p ΔCt values were higher in ***cfs0*** than ***sc0***, but the differences were not significant after ANOVA and Tukey tests.

### Post-exercise groups

**Figure 6:**
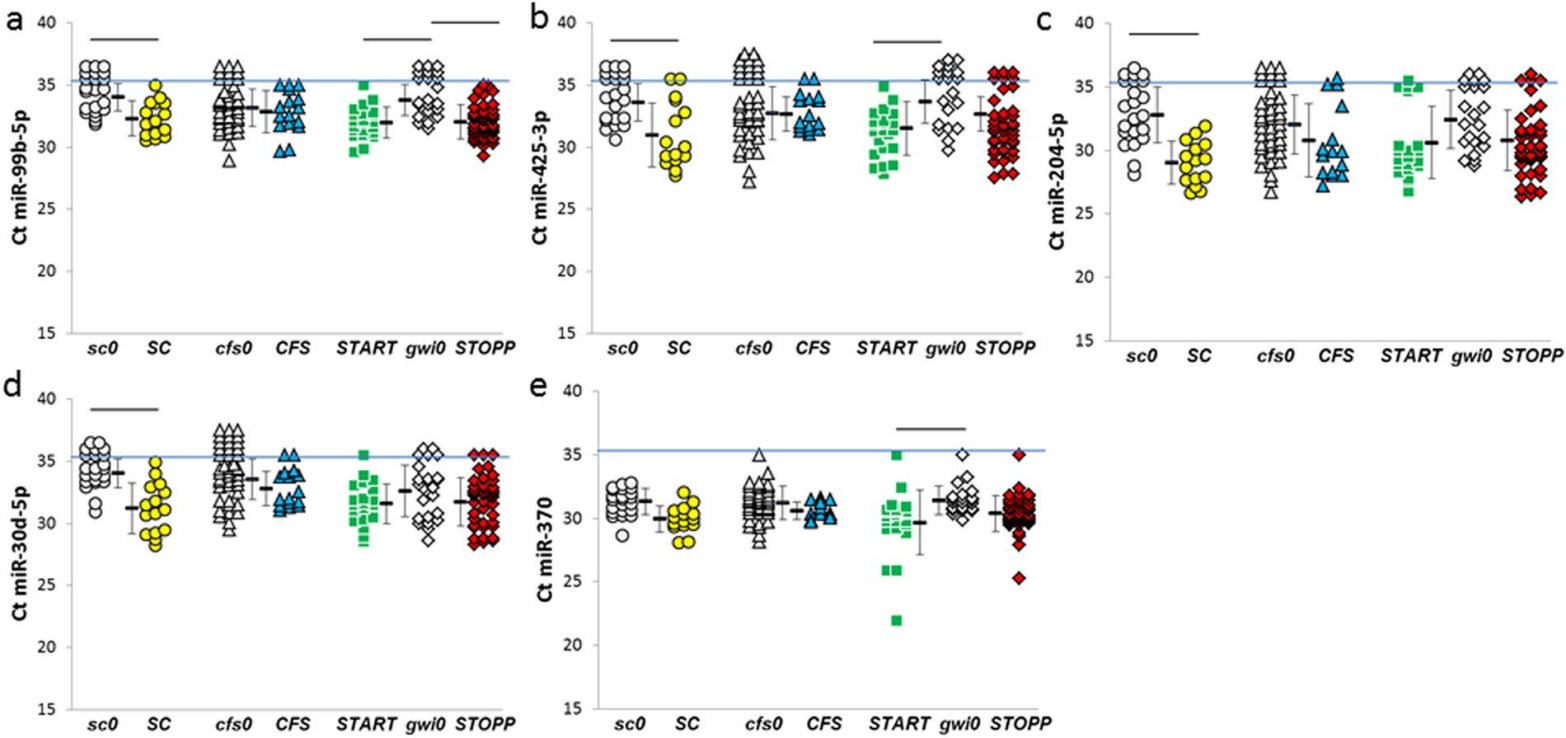
miRNA differences between post-exercise groups. (**a**) miR-22-3p was not detectable in most of the ***START*** subjects (green squares above the blue line at Ct = 35). ***START*** had significantly less miR-22-3p than ***SC*** (yellow circles) and ***STOPP*** (red diamonds) as indicated by bars over top of the groups (HSD < 0.05, FDR < 0.10). In addition, ***SC*** had significantly more miR-22-3p than ***sc0*** (grey circles). (**b**) miR-9-3p was detected in ***START***, but was found in fewer than two thirds of subjects in the other groups. ***START*** had significantly more miRNA expression than ***STOPP*** (ΔΔCt = 1.6 ± 1.4, mean ± SD, HSD < 0.05, FDR < 0.10).

miR-22-3p and miR-9-3p were the only miRNAs to be significantly different between the post-exercise groups (Fig. 6). miR-22-3p was an anomaly by having wide ranges of Ct in all groups. miR-22-3p was virtually not detectable in ***START***, and so levels in START were significantly diminished compared to ***SC*** and ***STOPP*** (Supplementary Table S6). The reduction in ***START***, but relative increase in ***STOPP***, supported the presence of 2 phenotypes of GWI. Specificities and sensitivities were 0.76 for ***START*** versus ***SC*** (Ct threshold of 29) and ***START*** versus ***STOPP*** (Ct threshold of 33) (Table 2).

miR-9-3p demonstrated a different trend. Only the ***START*** group had detectable levels (Ct ≤ 35) in more than two thirds of subjects. The difference between ***START*** and ***STOPP*** was small (Fig. 6, Supplementary Table S6) but significant (HSD < 0.05, FDR < 0.05). The low specificity and sensitivity of 65% at a threshold of Ct = 33 reflected the low levels of miR-9-3p in cerebrospinal fluid.

### miRNAs elevated after exercise compared to nonexercise groups

**Figure 7:**
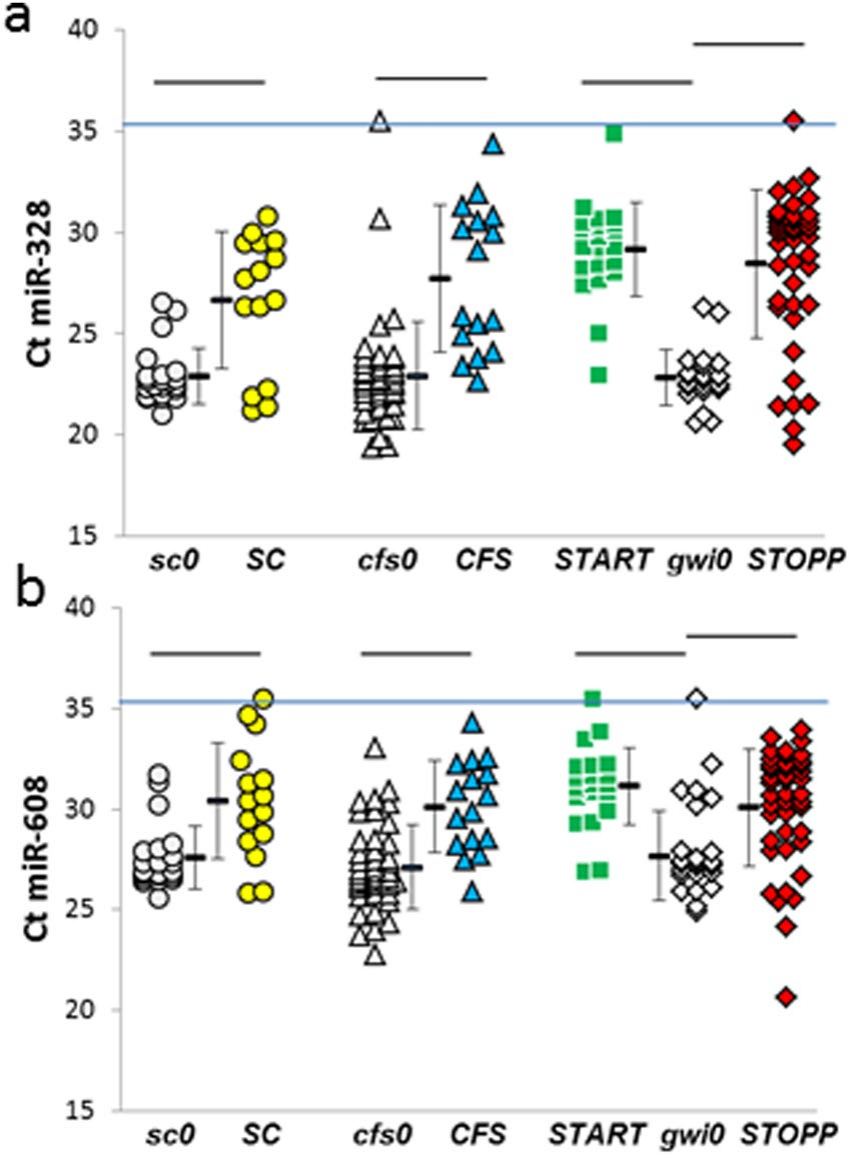
miRNAs that were significantly elevated in post-exercise compared to appropriate nonexercise control groups. Significant differences between groups were indicated by the bars at the top of the graphs for (**a**) miR-99b-5p, (**b**) miR-425-3p, (**c**) miR-30d-5p, (**d**) miR-204-5p, and (**e**) miR-370 (HSD ≤ 0.05, FDR ≤ 0.10, detected with Ct ≤ 35 in more than two thirds of one group per pair). The horizontal blue line indicated Ct = 35. Mean ± SD.

Exercise elevated the levels of several miRNAs compared to appropriate nonexercise groups. ***SC*** had higher levels than ***sc0*** for miR-22-3p (Fig. 6), miR-30d-5p, miR-204-5p, miR-425-3p, and miR-99b-5p (Fig. 7). Specificities and sensitivities for miR-204-5p and miR-22-3p were 0.80 at thresholds of 31 and 32, respectively.

***START*** had elevated levels of miR-425-3p and miR-99b-5p when compared to ***gwi0***. miR-370 was detected in almost all cerebrospinal fluid samples, but only ***START*** had a significant elevation compared to nonexercise (ΔΔCt = 1.7 ± 2.1 versus ***gwi0***, mean ± SD). ***STOPP*** shared the exercise – induced elevation of miR-99-5p with ***SC*** and ***START***. Specificities and sensitivities for miR-99b-5p were about 0.75 at Ct thresholds of 33 for ***SC***, ***START*** and ***STOPP*** compared to their nonexercise controls.

***CFS*** did not have any elevations of miRNA levels compared to ***cfs0***.

### miRNAs diminished after exercise compared to nonexercise groups

**Figure 8:**
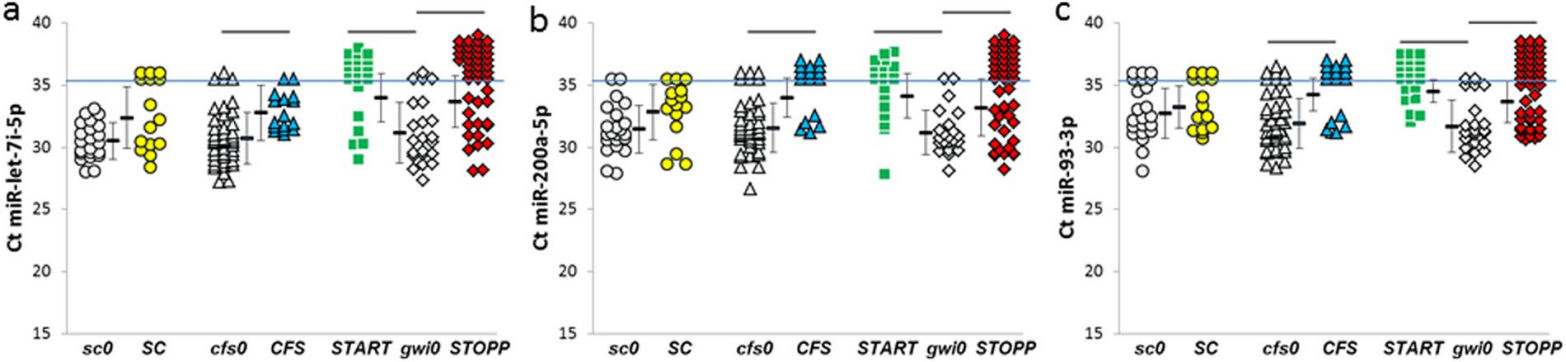
Decreased (**a**) miR-328 and (**b**) miR-608 levels in ***SC***, ***CFS***, ***START*** and ***STOPP*** groups after exercise.

miR-328 and miR-608 were significantly diminished by exercise in ***SC***, ***CFS***, ***START*** and ***STOPP*** compared to the nonexercise ***sc0***, ***cfs0***, and ***gwi0*** groups (Fig. 8). These miRNAs were detectable in almost all cerebrospinal fluid specimens in this study. Specificities and sensitivities for miR-328 ranged from 0.74 at Ct = 23 for ***SC***, 0.84 for ***CFS***, 0.86 for ***STOPP*** and 0.91 for ***START*** (Ct thresholds of 23 to 25). Specificities and sensitivities for miR-608 ranged from 0.78 to 0.83 (thresholds = 28). Diminished miR-328 and miR-608 may be a consequence of exercise that affected all subjects regardless of their disease status.

**Figure 9:**
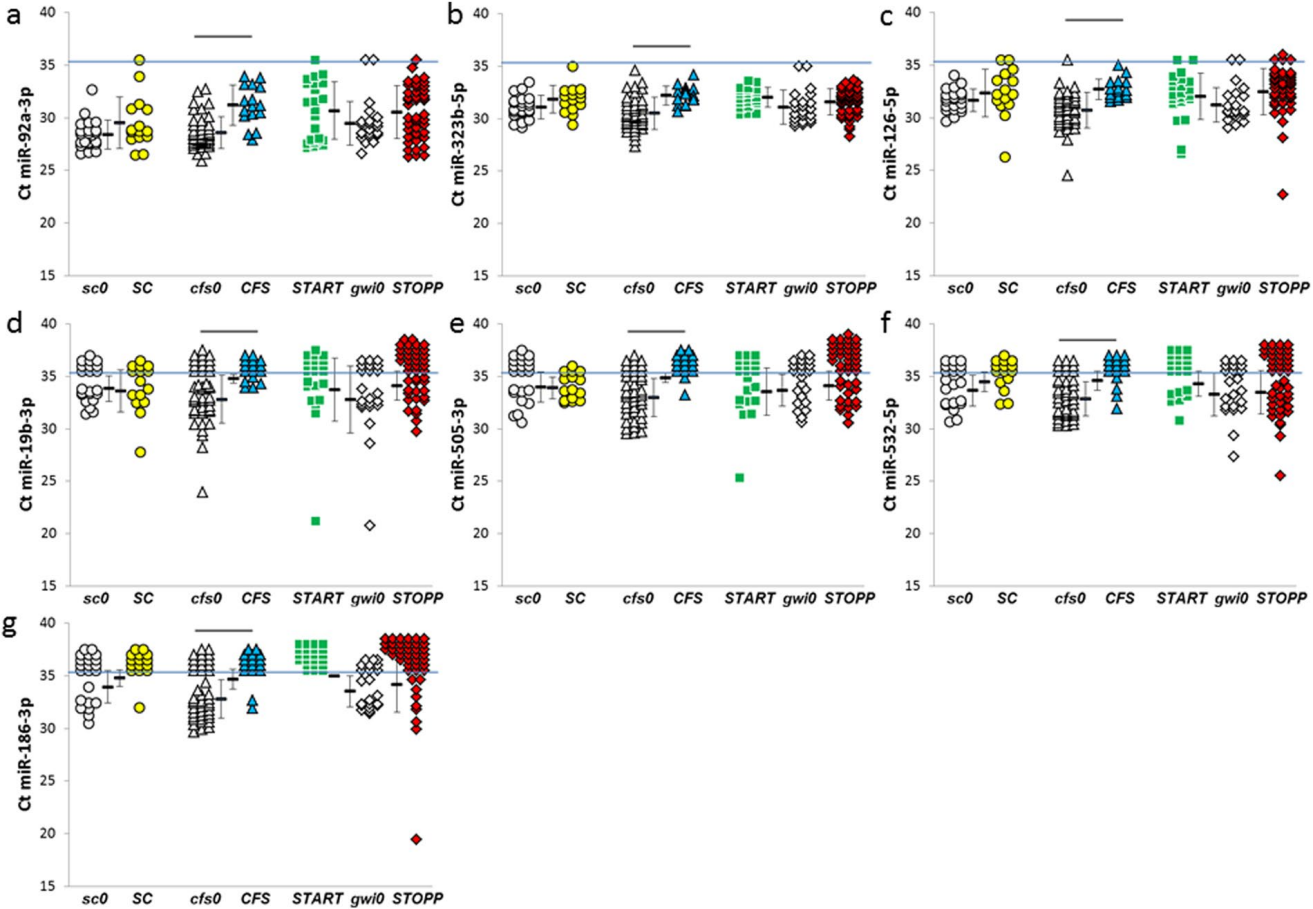
miRNAs reduced by exercise. GWI phenotypes (***START*** and ***STOPP***) and ***CFS*** all had reductions in (**a**) miR-let-7i-5p, (**b**) miR-200a-5p and (**c**) miR-93-3p. Sedentary controls had no changes.

miR-let-7i-5p, miR-200a-5p and miR-93-3p were significantly reduced in ***START***, ***STOPP*** and ***CFS*** compared to their ***gwi0*** and ***cfs0*** nonexercise controls (Fig. 9). They were unchanged between ***SC*** and ***sc0*** groups.

**Figure 10:**
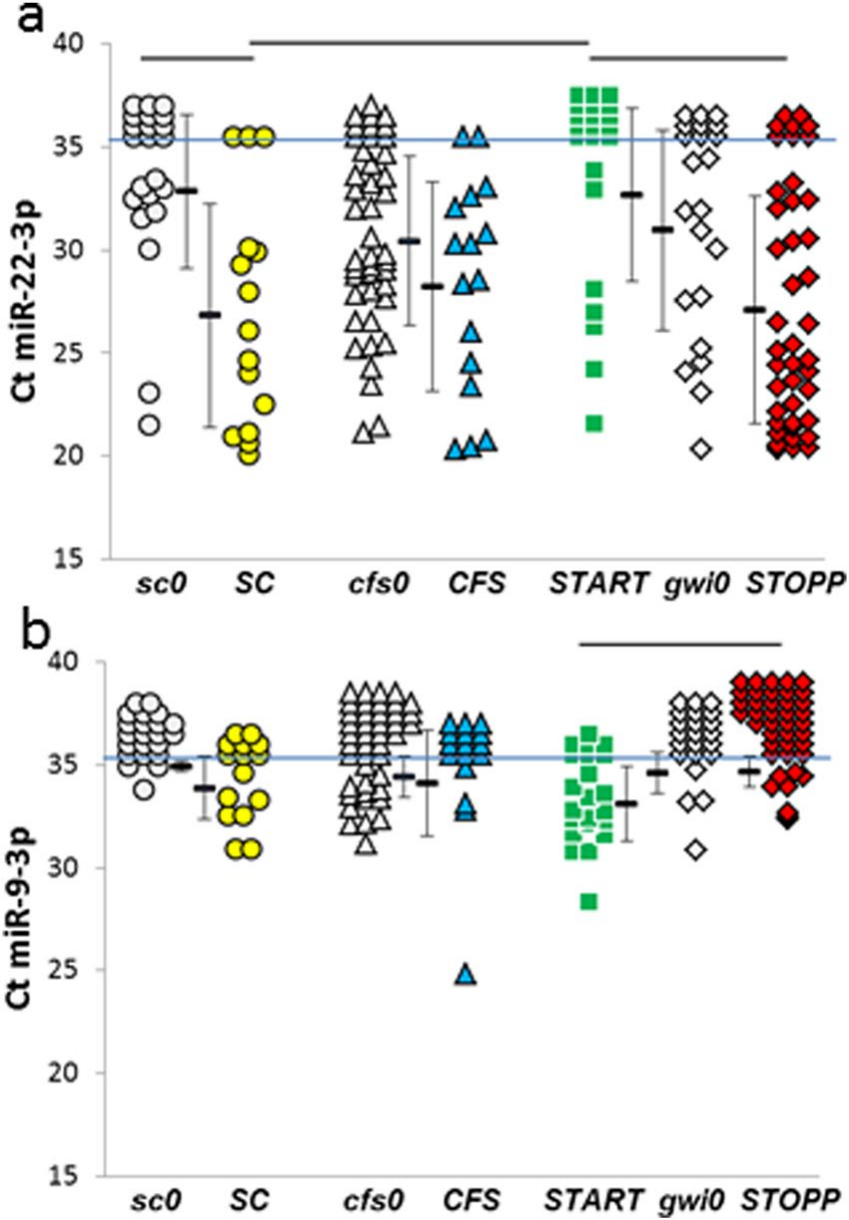
Decreased miRNAs after exercise in ***CFS*** group. ***CFS*** had significant reductions in -(**a**) 92a-3p, (**b**) miR-323b-5p, (**c**) miR-126-5p, (**d**) miR-19b-3p, (**e**) miR-505-3p, (**f**) miR-532-5p, and (**g**) miR-186-3p, compared to its nonexercise ***cfs0*** comparison group (bars above the groups).

***CFS*** was distinguished from the other groups by having significant reductions of miR-126-5p, miR-186-3p, miR-19b-3p, miR-92a-3p and miR-505-3p compared to the nonexercise ***cfs0*** group (Fig. 10). Specificities and sensitivities were about 0.82 for miR-328, miR-608 and miR-92a-3p. The large number of exercise – induced reductions in miRNAs differentiated ***CFS*** from ***SC*** and the GWI phenotypes.

### Gender

Cerebrospinal fluid miRNA levels for females and males in the nonexercise and the post-exercise groups were equivalent except for a significantly higher level of miR-9-3p in ***START*** than ***STOPP*** males (ΔΔCt = 1.7 ± 1.4)^43^.

The only exercise - induced change in females was a reduction in miR-328 in the ***STOPP*** group compared to ***gwi0*** (5.7 ± 0.8). Samples sizes for the post-exercise ***SC*** and ***START*** females (n = 3 each) were too small to infer meaningful differences.

The post-exercise control males had significantly elevated miR-204-3p (4.4 ± 2.8), miR-30d-5p (3.4 ± 2.3) and miR-30a-5p (2.9 ± 2.0) compared to nonexercise males.

miR-328 was reduced by exercise in ***START*** (7.2 ± 5.4), ***STOPP*** (6.9 ± 4.8) and ***CFS*** (5.7 ± 3.7) males compared to nonexercise males. Control males had a similar magnitude change that was not significant by FDR (3.3 ± 3.2). ***STOPP*** males had significantly diminished miR-608 (4.6 ± 3.2) and miR-200a-5p (3.7 ± 2.1).

These differences were consistent with the overall group effects

## Discussion

This is the first description of the effects of exercise on cerebrospinal fluid miRNA expression in healthy subjects. Exercise diminished miR-328 and miR-608 in all subjects suggesting a general effect on the brain (Fig. 8, Supplementary Table S5). Exercise caused distinct patterns of miRNA changes in ***CFS*** and the ***START*** and ***STOPP*** phenotypes of GWI indicating significant pathophysiological differences between conditions.

Unlike our starting hypothesis, there were no differences in miRNA levels between the nonexercise groups of control, CFS and GWI subjects. Therefore, baseline levels of cerebrospinal fluid miRNAs may not be useful for diagnosis of CFS or GWI.

The only significant differences between groups after exercise were diminished miR-22-3p in ***START*** compared to ***SC*** and ***STOPP***, and elevated miR-9-3p in ***START*** compared to ***STOPP*** (Fig. 6). These differences between ***START*** and ***STOPP*** support our 2 phenotypes of GWI^25^.

The most striking findings were the changes between post-exercise groups and their appropriate nonexercise comparison groups. ***SC*** had 5 elevated miRNAs after exercise, compared to 3 for ***START***, 1 for ***STOPP***, and none in ***CFS*** (Fig. 7, Supplementary Table S4).

The reduction of miR-608 after exercise has implications for the cholinergic hypothesis of GWI pathophysiology because it targets acetylcholinesterase and interleukin-6 (IL6) mRNAs. miR-608 binds weakly to the single-nucleotide polymorphism rs17228616 allele in the 3′-untranslated region of acetylcholinesterase mRNA^44^. Homozygotes for rs17228616 have reduced affinity for miR-608. This promotes mRNA stability and increases acetylcholinesterase protein translation. As a consequence, more miR-608 is available to bind to IL6 mRNA and reduces its translation. This allele also contributes to reduced cortisol and elevated blood pressure. Because rs17228616 promotes higher acetylcholinestase activity, it may be relatively protective against nerve agent and pyridostimine bromide exposure.

miR-let-7i-5p, miR-93-3p and miR-200a-5p were significantly diminished after exercise in ***START***, ***STOPP*** and ***CFS***, but not ***SC*** (Fig. 9). This was consistent with a cardinal finding in CFS and GWI: function may appear normal when rested, but will deteriorate after a physiological stressor^1–4,7,25,45,46^. miR-let-7i was reduced in plasma after exercise in athletes^47^. miR-let-7i has decreased expression in the prefrontal cortex of FSL rats in a model of depression^48^. IL6 is a target of miR-let-7i, and, as predicted, this cytokine was significantly elevated in the brains of these rats. When FSL rats were given access to running wheels, their miR-let-7i expression was increased and IL6 reduced. Modulation of miR-let-7i and IL-6 may contribute to exercise-induced benefits in “inflammatory” depression^48^. miR-let-7i also contributes to the regulation of acetylcholine’s muscarinic and α4β2 nicotinic receptors and epigenetic regulation of acetylcholinesterase. These animal models may not be appropriate for CFS or GWI because human subjects develop exertional exhaustion after exercise, and are unlikely to significantly increase spontaneous exercise levels when provided with a treadmill^45^.

The ***CFS*** group had 12 miRNAs reduced after exercise. miR-186-3p was decreased in aging mice^49^ where it targets β-site amyloid precursor protein-cleaving enzyme 1 (BACE1) mRNA. Diminished miR-186-3p allows increased BACE1 mRNA translation and cleavage of amyloid peptides that increase the risk for brain disease. miR-19b-3p was reduced in serum from Alzheimer’s patients, and targeted signal transduction and activator of transcription 3 (STAT3) mRNA in a murine model^50^. miR-92a-3p was increased in glioblastoma and targeted BCL2L11 to reduce tumor apoptosis^51^. Its reduction after exercise may promote apoptosis in CFS. miR-126-5p was highly expressed in endothelial cells where it targets vascular (VCAM), intercellular (ICAM) and activated leukocyte (ALCAM) cell adhesion molecule mRNAs and so reduces transendothelial migration^52,53^. This is relevant for immune cell influx into the brain, and hypotheses of neuroinflammation in CFS pathogenesis.

Neurons may be the sources of miR-124-3p, miR-127-3p, miR-433, and miR-323b-5p (Figs 4 and 10)^54^. There was little overlap with the miRNAs synthesized in astrocytes, oligodendrocytes and microglia.

The choroid plexus epithelium may be a primary source of miRNAs in cerebrospinal fluid^55–57^. Epithelial cells form a monolayer linked by tight junctions that creates the “shrink wrapped” cellular barrier around fenestrated capillaries^58^. Interferon-gamma and other mediators generated by exercise, inflammation, and other stressors act directly on choroid plexus to modulate barrier permeability, plasma protein transport, protein synthesis and secretion of nutrients into cerebrospinal fluid^59–61^. miR-328, which was present in all subjects and reduced after exercise (Fig. 8), binds to the 3′-untranslated regions of CD44 and collagen type 1α1 mRNAs to modulate extracellular barrier functions^62^. Choroid plexus miRNAs^55^ are packaged into extracellular vesicles and released into cerebrospinal fluid^56,57,63,64^. Downstream targets include subventricular neural stem cells, mature neurons, astrocytes, oligodendrocytes, microglia, meningeal and central immune cells^56,57,63,65–67^. Blockade of extracellular vesicle secretion from choroid plexus cells decreased brain inflammation in a mouse model of lipopolysaccharide-induced inflammation^56^. Choroid plexus miRNAs may be novel drug targets to modulate acute illness behaviours, fever, and chronic pain in systemic illnesses.

Choroid plexus is dysfunctional in Alzheimer’s disease^68^. This provides the rationale to consider the role of the blood – cerebrospinal fluid barrier in the cognitive dysfunction of CFS and GWI. There are numerous reports of elevated and diminished miRNAs in cerebrospinal fluid in Alzheimer’s disease^36,69–74^, but none matched the patterns of our groups. miR-let-7i-5p^36^ was elevated in Alzheimer’s, but levels were equivalent in nonexercise groups (Fig. 8).

Depression is in the differential diagnosis because of the shared ancillary diagnostic criteria (Fig. 1)^75–78^. Major depressive disorder is defined by affective dysfunction with sadness, flat affect and anhedonia as essential features, followed by secondary criteria including fatigue, cognitive, sleep, and somatic dysfunction^78^. However, screening questionnaires for depression emphasize somatic symptoms^79,80^. Complaints of fatigue, sleep, and cognitive dysfunction will inflate total questionnaire scores, and may lead to false positive inference of major depressive disorder even if anhedonia or affective complaints are absent^81–83^. This is particularly problematic in CFS and GWI where these features are diagnostic criteria (Fig. 1). As a result, Center for Epidemiology – Depression (CESD)^33^ scores were significantly elevated in GWI (78.3%), CFS (64.0%) and control (25.0%) groups (Table 1).

Quantitative PCR of miRNAs offers a more objective solution^84^. miR-16 in cerebrospinal fluid was significantly lower in major depressive disorder patients than control subjects^85^. However, this was not confirmed in an independent group who had a different pattern of 11 significantly elevated and 5 reduced miRNAs^86^. Our data did not confirm either of these findings because only 3 of the miRNAs were detected with Ct ≤ 35 in more than two thirds of our nonexercise group. miR-425-3p was significantly reduced in depression patients^84–86^, and was detected in about half of all nonexercise subjects. It was increased after exercise in ***SC***, ***START*** and ***STOPP*** but not ***CFS*** (Fig. 7). This lack of reproducibility highlights the need to independently verify miRNA findings, and supports our rationale for strict statistical criteria to define potential miRNA biomarkers.

The pain and tenderness of GWI subjects (Table 1) indicated systemic hyperalgesia and suggested parallels with fibromyalgia^34^. Nine miRNAs were virtually undetectable in 10 fibromyalgia women compared to 8 healthy control women^87^. miR-99b-5p and miR-29a-3p were absent in fibromyalgia, but were detected in more than two thirds of our participants. miR-99b-5p was significantly increased after exercise in ***SC***, ***START*** and ***STOPP*** (Fig. 7). The other 7 miRNAs were detected in less than half of our specimens. This suggested that GWI and CFS were distinct from fibromyalgia.

Limitations to the diagnostic use of quantitative miRNA analysis in cerebrospinal fluid include the remarkable lack of consensus about miRNA levels in control subjects. This can be remedied by standardization of reagents and protocols^72^, open source sharing of study outcomes, and meta-analysis of the raw data. The yield of extracted miRNA^88^ and detectability were improved with 0.5 ml instead of 0.2 ml of cerebrospinal fluid^36,87^. QPCR with Ct cut-offs ≤ 35 cycles reduced amplification artifacts^73,87^. The wide range of miR-22-3p Ct values (Fig. 6) may be due to commercial changes in reagents designed to improve miRNA detection. Highly abundant miRNAs that were detected with Ct < 35 in all subjects were used as normalizers (Fig. 4) rather than the global average level, miR-423-5p, miR-124-3p or U6^36,71,73,86,89^. Constraints included (i) significant ANOVA and Tukey HSD between groups, (ii) significant FDR to correct for multiple comparisons, and (iii) focusing on miRNAs that were detected in more than two thirds of subjects per group that may be viable biomarker candidates for use in the general population^36,71,86^. Ages were comparable between groups^74^ (Table 1) and there were no differences in expression between males and females^43,87^. Next generation sequencing is an excellent discovery tool but needs careful internal standardization to be as sensitive as QPCR for quantification^90–92^. Adequate sample sizes were essential because our initial findings with about a dozen subjects per group showed differences between ***START*** and ***STOPP*** after exercise^93^, but these differences eventually regressed to the mean as more subjects were analyzed. This is especially pertinent to smaller studies examining the differential diagnosis of CFS and GWI^85–87,94,95^.

Limitations of the testing paradigm include the intensive nature of the exercise and magnetic resonance imaging characterization of GWI subjects to determine their phenotypes. Lumbar puncture was required to obtain the cerebrospinal fluid miRNA biomarkers, but this procedure is not a contraindication to making an objective diagnosis of GWI. On the contrary, magnetic resonance imaging with cerebrospinal fluid QPCR miRNA profiling may be complementary tools for diagnosis of CFS, GWI and their subtypes.

## Conclusions

Cerebrospinal fluid miRNA levels were equivalent between SC, CFS and GWI subjects who had rested before exercise (nonexercise groups). miRNA levels were different from the ones that are altered in depression, fibromyalgia, and Alzheimer’s disease suggesting that these are all distinct diseases, or that the data from those smaller studies could not be replicated in this larger study. miRNA levels were equivalent between the post-exercise ***SC***, ***CFS*** and GWI phenotypes of ***START*** and ***STOPP*** with the exception of miR-22-3p and miR-9-3p that significantly distinguished ***START*** from ***STOPP***. This adds another layer of evidence to support neurotoxic pathology in GWI^27^ and these 2 phenotypes of GWI veterans^25^. Post-exercise levels were significantly elevated (n = 6) or diminished (n = 12) compared to the nonexercise comparison groups. miR-328 and miR-608 were elevated in ***SC***, ***CFS***, ***START*** and ***STOPP*** and may be a global marker of the exercise stressor on the choroid plexus and brain. ***CFS*** had 12 diminished and zero elevated miRNAs after exercise indicating its pathophysiology and responses to exercise were unique compared to GWI and control subjects. Despite the symptom overlap of CFS, GWI and other illnesses in the differential diagnosis (Fig. 1), the distinct exercise-induced miRNA patterns in cerebrospinal fluid imply separate mechanisms for post-exertional malaise in these diseases.

## Electronic supplementary material

SOM Table 1

SOM Tables 2,4,5,6

SOM Table 3

## References

[1] Fukuda K (1994). The chronic fatigue syndrome: a comprehensive approach to its definition and study. International Chronic Fatigue Syndrome Study Group. Ann. Intern. Med..

[2] Carruthers BM (2003). Myalgic Encephalomyelitis/chronic fatigue syndrome: Clinical working case definition, diagnostic and treatments protocols. J. C. F. S..

[3] Carruthers BM (2011). Myalgic Encephalomyelitis: International Consensus Criteria. J. Intern. Med..

[4] Beyond Myalgic Encephalomyelitis/Chronic Fatigue Syndrome: Redefining an Illness. (Institute of Medicine, 2015).

[5] Centers for Disease Control and Prevention (CDC). Unexplained illness among Persian Gulf War veterans in an Air National Guard Unit: preliminary report–August 1990-March 1995. M.*M*.*W*.*R*. *Morb*. *Mortal*. *Wkly*. *Rep*. 4**4**, 443–7 (1995). 7770029

[6] Fukuda K (1998). Chronic multisymptom illness affecting Air Force veterans of the Gulf War. J.A.M.A..

[7] Steele L (2000). Prevalence and patterns of Gulf War illness in Kansas veterans: association of symptoms with characteristics of person, place, and time of military service. Am. J. Epidemiol..

[8] Gulf War Illness and the Health of Gulf War Veterans, **57** 29–30 (Research Advisory Committee on Gulf War Veterans’ Illnesses, 2008).

[9] http://iacfsme.org/ME-CFS-Primer-Education/News/News-Related-Docs/2014/ME-CFS-Primer-for-Clinical-Practitioners-(2014-rev.aspx. Accessed January 2, 2017.

[10] Greenberg DB (1990). Neurasthenia in the 1980s: chronic mononucleosis, chronic fatigue syndrome, and anxiety and depressive disorders. Psychosomatics..

[11] Holmes GP (1987). A cluster of patients with a chronic mononucleosis-like syndrome. Is Epstein-Barr virus the cause?. J.A.M.A..

[12] Roy MJ, Koslowe PA, Kroenke K, Magruder C (1998). Signs, symptoms, and ill-defined conditions in Persian Gulf War veterans: findings from the Comprehensive Clinical Evaluation Program. Psychosom. Med..

[13] Naviaux RK (2016). Metabolic features of chronic fatigue syndrome. Proc. Natl. Acad. Sci. USA.

[14] Fluge Ø (2016). Metabolic profiling indicates impaired pyruvate dehydrogenase function in myalgic encephalopathy/chronic fatigue syndrome. J.C.I. Insight..

[15] Barnden LR (2015). Evidence in chronic fatigue syndrome for severity-dependent upregulation of prefrontal myelination that is independent of anxiety and depression. N.M.R. Biomed..

[16] Barnden LR (2016). Autonomic correlations with MRI are abnormal in the brainstem vasomotor centre in Chronic Fatigue Syndrome. Neuroimage Clin..

[17] Iacob E (2016). Gene Expression Factor Analysis to Differentiate Pathways Linked to Fibromyalgia, Chronic Fatigue Syndrome, and Depression in a Diverse Patient Sample. Arthritis Care Res. (Hoboken)..

[18] Abdullah L (2016). Translational potential of long-term decreases in mitochondrial lipids in a mouse model of Gulf War Illness. Toxicology.

[19] Steele L (2012). Complex factors in the etiology of Gulf War illness: wartime exposures and risk factors in veteran subgroups. Environ. Health Perspect..

[20] Baraniuk JN (2005). A Chronic Fatigue Syndrome - related proteome in human cerebrospinal fluid. BMC Neurol..

[21] Steele L (2015). Butyrylcholinesterase genotype and enzyme activity in relation to Gulf War illness: preliminary evidence of gene-exposure interaction from a case-control study of 1991 Gulf War veterans. Environ. Health.

[22] Rayhan RU (2013). Increased brain white matter axial diffusivity associated with fatigue, pain and hyperalgesia in Gulf War illness. PLoS One.

[23] Chao LL (2011). Effects of low-level sarin and cyclosarin exposure and Gulf War Illness on brain structure and function: a study at 4T. Neurotoxicology.

[24] Koslik HJ, Hamilton G, Golomb BA (2014). Mitochondrial dysfunction in Gulf War illness revealed by 31Phosphorus Magnetic Resonance Spectroscopy: a case-control study. PLoS One..

[25] Rayhan RU (2013). Exercise challenge in Gulf War Illness reveals two subgroups with altered brain structure and function. PLoS One.

[26] Liaw MY (1998). Central representation of phantom limb phenomenon in amputees studied with single photon emission computerized tomography. Am. J. Phys. Med. Rehabil..

[27] White RF (2016). Recent research on Gulf War illness and other health problems in veterans of the 1991 Gulf War: Effects of toxicant exposures during deployment. Cortex..

[28] Krol J, Loedige I, Filipowicz W (2010). The widespread regulation of microRNA biogenesis, function and decay. Nat. Rev. Genet..

[29] Baraniuk JN (2013). A Chronic Fatigue Syndrome (CFS) severity score based on case designation criteria. Am. J. Transl. Res..

[30] Baraniuk JN, Clauw DJ, Gaumond E (1998). Rhinitis symptoms in chronic fatigue syndrome. Ann. Allergy Asthma Immunol..

[31] Ware JE, Sherbourne CD (1992). The MOS 36-item short-form health survey (SF-36). I. Conceptual framework and item selection. Med. Care..

[32] Löwe B (2008). Validation and standardization of the Generalized Anxiety Disorder Screener (GAD-7) in the general population. Med. Care..

[33] Roberts RE, Vernon SW (1983). The Center for Epidemiologic Studies Depression Scale: its use in a community sample. Am. J. Psychiatry.

[34] Wolfe F (1990). The American College of Rheumatology 1990 Criteria for the Classification of Fibromyalgia. Report of the Multicenter Criteria Committee. Arthritis Rheum..

[35] Light AR (2012). Gene expression alterations at baseline and following moderate exercise in patients with Chronic Fatigue Syndrome and Fibromyalgia Syndrome. J. Intern. Med..

[36] Sørensen SS, Nygaard AB, Christensen T (2016). miRNA expression profiles in cerebrospinal fluid and blood of patients with Alzheimer’s disease and other types of dementia - an exploratory study. Transl. Neurodegener..

[37] Shivapurkar N (2014). Recurrence of early stage colon cancer predicted by expression pattern of circulating microRNAs. PLoS One..

[38] LaConti. JJ (2011). Tissue and serum microRNAs in the Kras(G12D) transgenic animal model and in patients with pancreatic cancer. PLoS One.

[39] Shivapurkar N (2014). Decrease in blood miR-296 predicts chemotherapy resistance and poor clinical outcome in patients receiving systemic chemotherapy for metastatic colon cancer. Int. J. Colorectal Dis..

[40] Livak KJ, Schmittgen TD (2001). Analysis of relative gene expression data using real-time quantitative PCR and the Delta Delta C(t) method. Methods..

[41] Benjamini Y, Hochberg Y (1995). Controlling the False Discovery Rate: A practical and powerful approach to multiple testing. J. *Royal Stat*. Soc. B (Methodological)..

[42] Cohen, J. Statistical power analysis for the behavioral sciences. (2nd ed.). (Lawrence Earlbaum Associates. 1988).

[43] Smylie AL (2013). A comparison of sex-specific immune signatures in Gulf War illness and chronic fatigue syndrome. BMC Immunol..

[44] Hanin G (2014). Competing targets of microRNA-608 affect anxiety and hypertension. Human Molecular Genetics..

[45] Keller BA, Pryor JL, Giloteaux L (2014). Inability of myalgic encephalomyelitis/chronic fatigue syndrome patients to reproduce VO_2_peak indicates functional impairment. J. Transl. Med..

[46] Rayhan RU (2013). Prefrontal lactate predicts exercise-induced cognitive dysfunction in Gulf War Illness. Am. J. Transl. Res..

[47] Nielsen S (2014). The miRNA plasma signature in response to acute aerobic exercise and endurance training. PLoS One.

[48] Wei YB (2016). Elevation of Il6 is associated with disturbed let-7 biogenesis in a genetic model of depression. Transl Psychiatry..

[49] Kim J (2016). miR-186 is decreased in aged brain and suppresses BACE1 expression. J. Neurochem..

[50] Wu Y (2017). Lower Serum Levels of miR-29c-3p and miR-19b-3p as Biomarkers for Alzheimer’s Disease. Tohoku J. Exp. Med..

[51] Niu H (2012). miR-92a is a critical regulator of the apoptosis pathway in glioblastoma with inverse expression of BCL2L11. Oncol. Rep..

[52] Cayrol R (2008). Activated leukocyte cell adhesion molecule promotes leukocyte trafficking into the central nervous system. Nat. Immunol..

[53] Harris TA (2008). MicroRNA-126 regulates endothelial expression of vascular cell adhesion molecule 1. Proc. Natl. Acad. Sci. USA.

[54] Jovičić. A (2013). Comprehensive expression analyses of neural cell-type- specific miRNAs identify new determinants of the specification and maintenance of neuronal phenotypes. J. Neurosci..

[55] Deo M, Yu JY, Chung KH, Tippens M, Turner DL (2006). Detection of mammalian microRNA expression by *in situ* hybridization with RNA oligonucleotides. Dev. Dyn..

[56] Balusu S (2016). Identification of a novel mechanism of blood-brain communication during peripheral inflammation via choroid plexus-derived extracellular vesicles. E.M.B.O. Mol. Med..

[57] Falcão AM (2012). The path from the choroid plexus to the subventricular zone: go with the flow!. Front. Cell. Neurosci..

[58] Keep RF, Jones HC (1990). A morphometric study on the development of the lateral ventricle choroid plexus, choroid plexus capillaries and ventricular ependyma in the rat. Brain Res. Dev. Brain. Res..

[59] Nilsson C, Lindvall-Axelsson M, Owman C (1992). Neuroendocrine regulatory mechanisms in the choroid plexus-cerebrospinal fluid system. Brain Res. Brain Res. Rev..

[60] Marques F (2009). The choroid plexus response to a repeated peripheral inflammatory stimulus. BMC Neurosci..

[61] Marques F (2009). Kinetic profile of the transcriptome changes induced in the choroid plexus by peripheral inflammation. J. Cereb. Blood Flow Metab..

[62] Rutnam ZJ, Yang BB (2012). The non-coding 3′ UTR of CD44 induces metastasis by regulating extracellular matrix functions. J. Cell Sci..

[63] Bátiz LF (2016). Exosomes as novel regulators of adult neurogenic niches. Front. Cell Neurosci..

[64] Akers JC (2015). miRNA contents of cerebrospinal fluid extracellular vesicles in glioblastoma patients. J Neurooncol..

[65] Doetsch F (1999). Subventricular zone astrocytes are neural stem cells in the adult mammalian brain. Cell.

[66] Fuentealba LC, Obernier K, Alvarez-Buylla A (2012). Adult neural stem cells bridge their niche. Cell. Stem Cell..

[67] Baruch K, Schwartz M (2013). CNS-specific T cells shape brain function via the choroid plexus. Brain Behav. Immun..

[68] Bergen AA (2015). Gene expression and functional annotation of human choroid plexus epithelium failure in Alzheimer’s disease. BMC Genomics..

[69] Alexandrov PN (2012). microRNA (miRNA) speciation in Alzheimer’s disease (AD) cerebrospinal fluid (CSF) and extracellular fluid (ECF). Int. J. Biochem. Mol. Biol..

[70] Dangla-Valls, A. *et al*. CSF microRNA profiling in Alzheimer’s Disease: a screening and validation study. *Mol*. *Neurobiol*. 2016 Oct 13. [Epub ahead of print]. 10.1007/s12035-016-0106-x 27738874

[71] Kiko T (2014). MicroRNAs in plasma and cerebrospinal fluid as potential markers for Alzheimer’s disease. J. Alzheimers Dis..

[72] Müller M (2016). MicroRNA-29a is a candidate biomarker for Alzheimer’s disease in cell-free cerebrospinal fluid. Mol. Neurobiol..

[73] Denk J (2015). MicroRNA profiling of CSF reveals potential biomarkers to detect Alzheimer’s disease. PLoS One.

[74] van Harten AC, Mulders J, Scheltens P, van der Flier WM, Oudejans CB (2015). Differential expression of microRNA in cerebrospinal fluid as a potential novel biomarker for Alzheimer’s disease. J. Alzheimers Dis..

[75] Blore JD, Sim MR, Forbes AB, Creamer MC, Kelsall HL (2015). Depression in Gulf War veterans: a systematic review and meta-analysis. Psychol. Med..

[76] Stimpson NJ, Thomas HV, Weightman AL, Dunstan F, Lewis G (2003). Psychiatric disorder in veterans of the Persian Gulf War of 1991. Systematic review. Br. J. Psychiatry..

[77] Valero S (2013). The role of neuroticism, perfectionism and depression in chronic fatigue syndrome. A structural equation modeling approach. Compr. Psychiatry..

[78] Diagnostic and Statistical Manual of Mental Disorders (DSM-IV). 4th ed. (American Psychiatric Association, Washington, DC, 1994).

[79] McKenzie DP (2015). Developing a brief depression screen and identifying associations with comorbid physical and psychological illness in Australian Gulf War veterans. J. Psychosom. Res..

[80] Shafer AB (2006). Meta-analysis of the factor structures of four depression questionnaires: Beck, CES-D, Hamilton, and Zung. J. Clin. Psychol..

[81] Clarke DM, McKenzie DP (1994). A caution on the use of cut-points applied to screening instruments or diagnostic criteria. J. Psychiatr. Res..

[82] Nanthakumar S, Bucks RS, Skinner TC (2016). Are we overestimating the prevalence of depression in chronic illness using questionnaires? Meta-analytic evidence in obstructive sleep apnoea. Health Psychol..

[83] Pincus T, Hassett AL, Callahan LF (2009). Criterion contamination of depression scales in patients with rheumatoid arthritis: the need for interpretation of patient questionnaires (as all clinical measures) in the context of all information about the patient. Rheum. Dis. Clin. North. Am..

[84] Dwivedi Y (2016). Pathogenetic and therapeutic applications of microRNAs in major depressive disorder. Prog. Neuropsychopharmacol. Biol. Psychiatry..

[85] Song MF (2015). CSF miR-16 is decreased in major depression patients and its neutralization in rats induces depression-like behaviors via a serotonin transmitter system. J. Affect. Disord..

[86] Wan Y (2015). Identification of differential microRNAs in cerebrospinal fluid and serum of patients with major depressive disorder. PLoS One..

[87] Bjersing JL (2013). Profile of cerebrospinal microRNAs in fibromyalgia. PLoS One.

[88] McAlexander MA, Phillips MJ, Witwer KW (2013). Comparison of methods for miRNA extraction from plasma and quantitative recovery of RNA from cerebrospinal fluid. Front. Genet..

[89] Andersen. CL, Jensen JL, Ørntoft TF (2004). Normalization of real-time quantitative reverse transcription-PCR data: a model-based variance estimation approach to identify genes suited for normalization, applied to bladder and colon cancer data sets. Cancer Res..

[90] Burgos K (2014). Profiles of extracellular miRNA in cerebrospinal fluid and serum from patients with Alzheimer’s and Parkinson’s diseases correlate with disease status and features of pathology. PLoS One..

[91] Nassirpour R (2014). Identification of tubular injury microRNA biomarkers in urine: comparison of next-generation sequencing and qPCR-based profiling platforms. BMC Genomics.

[92] Kuner R, Brase JC, Sültmann H, Wuttig D (2013). microRNA biomarkers in body fluids of prostate cancer patients. Methods.

[93] Baraniuk, J. N. & Shivapurkar, N. Cerebrospinal fluid MicroRNA (miRNA) in CFS and Gulf War Illness (GWI). International Association for Chronic Fatigue Syndrome/Myalgic Encephalomyelitis (IACFSME). Fort Lauderdale FLA, Oct. 2016.

[94] Brenu EW (2014). High-throughput sequencing of plasma microRNA in chronic fatigue syndrome/myalgic encephalomyelitis. PLoS One..

[95] Lopez JP (2017). MicroRNAs 146a/b-5 and 425-3p and 24-3p are markers of antidepressant response and regulate MAPK/Wnt-system genes. Nat. Commun..

