# Author Correction: Exercise – induced changes in cerebrospinal fluid miRNAs in Gulf War Illness, Chronic Fatigue Syndrome and sedentary control subjects

**DOI:** 10.1038/s41598-018-23238-0

**Published:** 2018-04-19

**Authors:** James N. Baraniuk, Narayan Shivapurkar

**Affiliations:** Division of Rheumatology, Immunology and Allergy, Department of Medicine, Georgetown University, Washington, District of Columbia United States of America

Correction to: *Scientific Reports* 10.1038/s41598-017-15383-9, published online 10 November 2017

This Article contains a low resolution version of Figure 2. A higher resolution image appears below as Figure 1.

**Figure 1:**
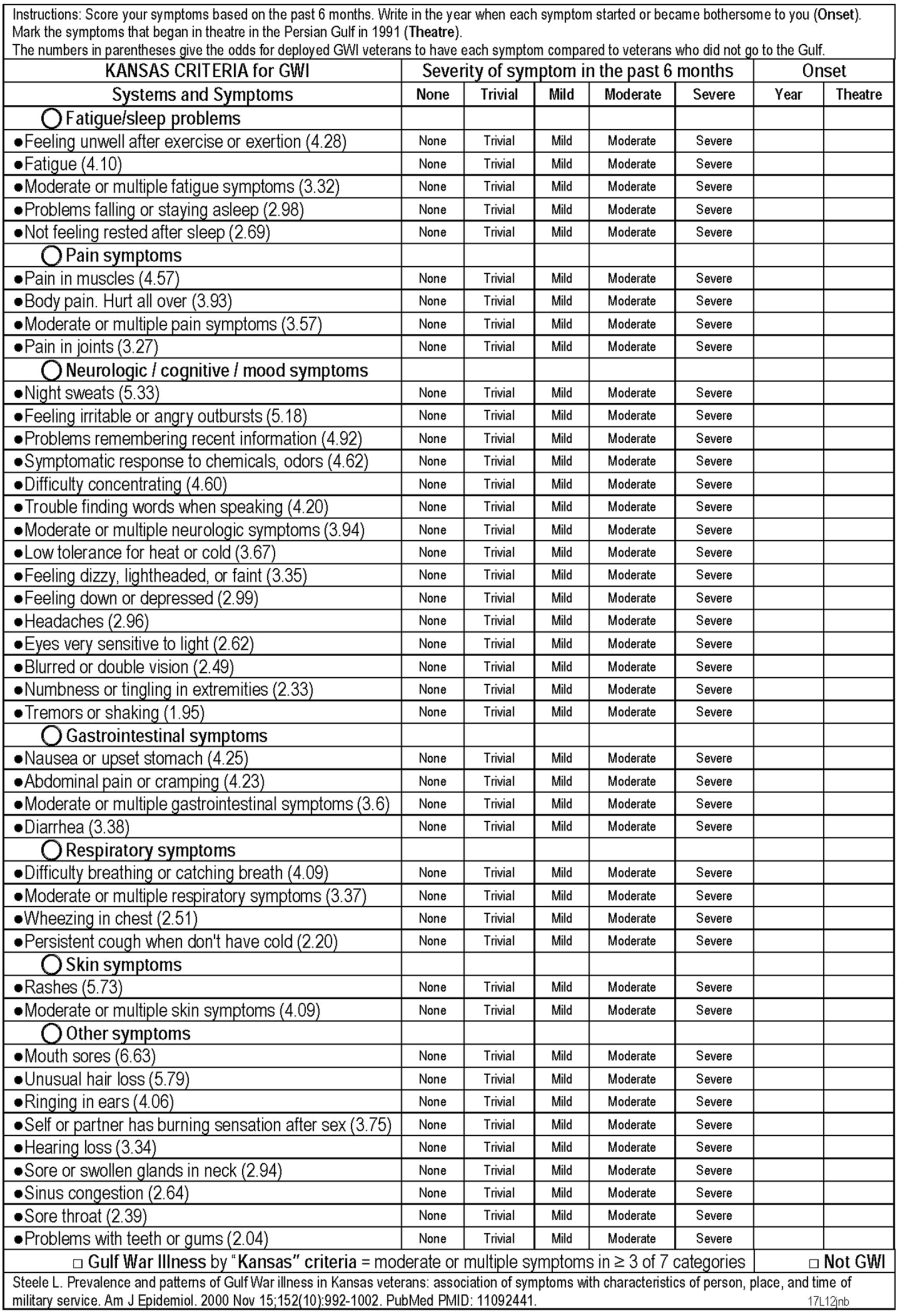
Kansas Criteria for Gulf War Illness scoring form based on Steele^7^. ©*JNBaraniukMD_17g13*. *Used with permission of the copyright holder*.

In addition, this Article contains an error in the order of the Figures. Figures 6, 7, 8, 9 and 10 were published as Figures 10, 6, 7, 8 and 9 respectively. The correct Figures 6, 7, 8, 9 and 10 appear below as Figures 2, 3, 4, 5 and 6 respectively. The Figure legends are correct.

**Figure 2:**
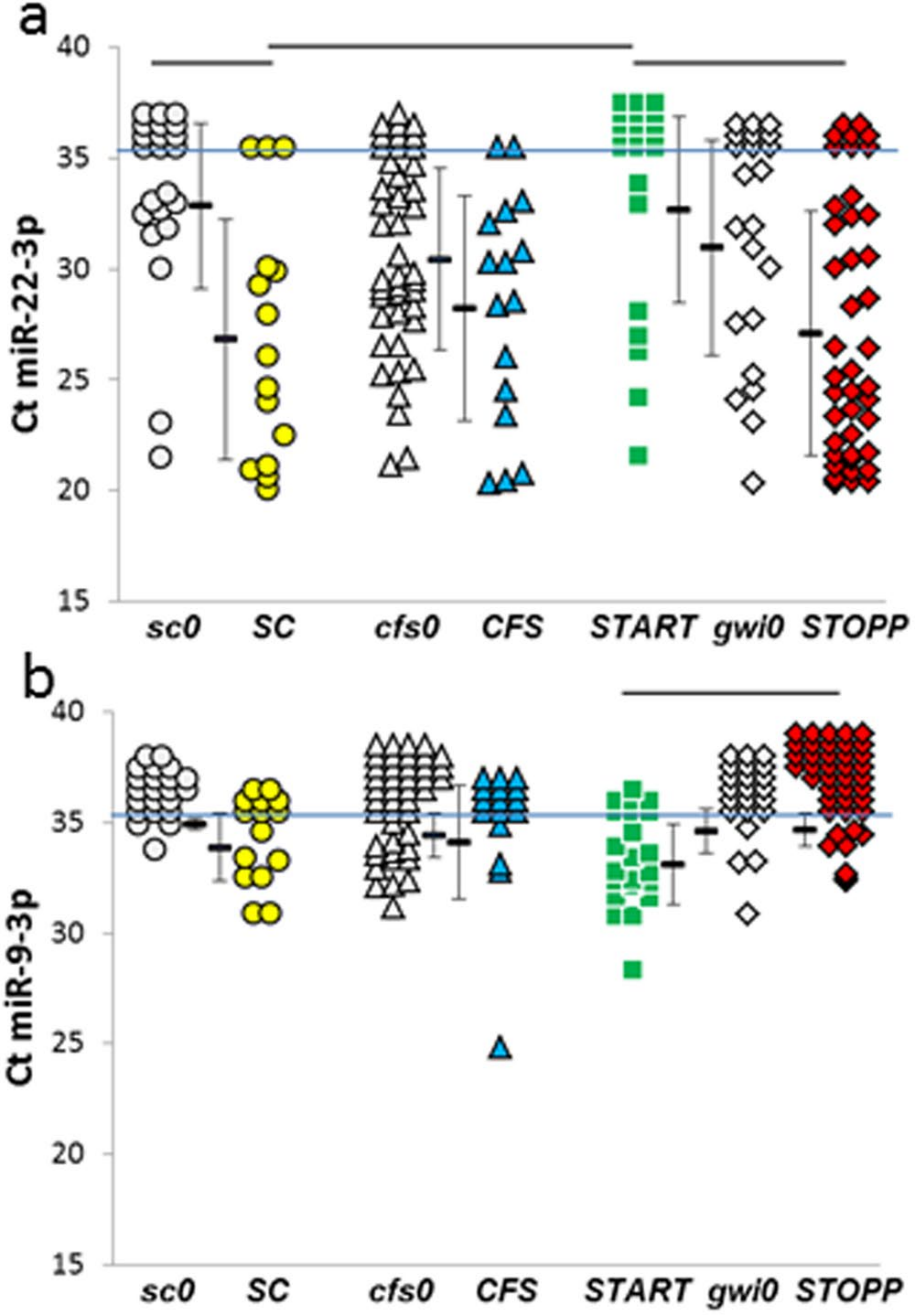
miRNA differences between post-exercise groups. (**a**) miR-22-3p was not detectable in most of the ***START*** subjects (green squares above the blue line at Ct = 35). ***START*** had significantly less miR-22-3p than ***SC*** (yellow circles) and ***STOPP*** (red diamonds) as indicated by bars over top of the groups (HSD < 0.05, FDR < 0.10). In addition, ***SC*** had significantly more miR-22-3p than ***sc0*** (grey circles). (**b**) miR-9-3p was detected in ***START***, but was found in fewer than two thirds of subjects in the other groups. ***START*** had significantly more miRNA expression than ***STOPP*** (ΔΔCt = 1.6 ± 1.4, mean ± SD, HSD < 0.05, FDR < 0.10).

**Figure 3:**
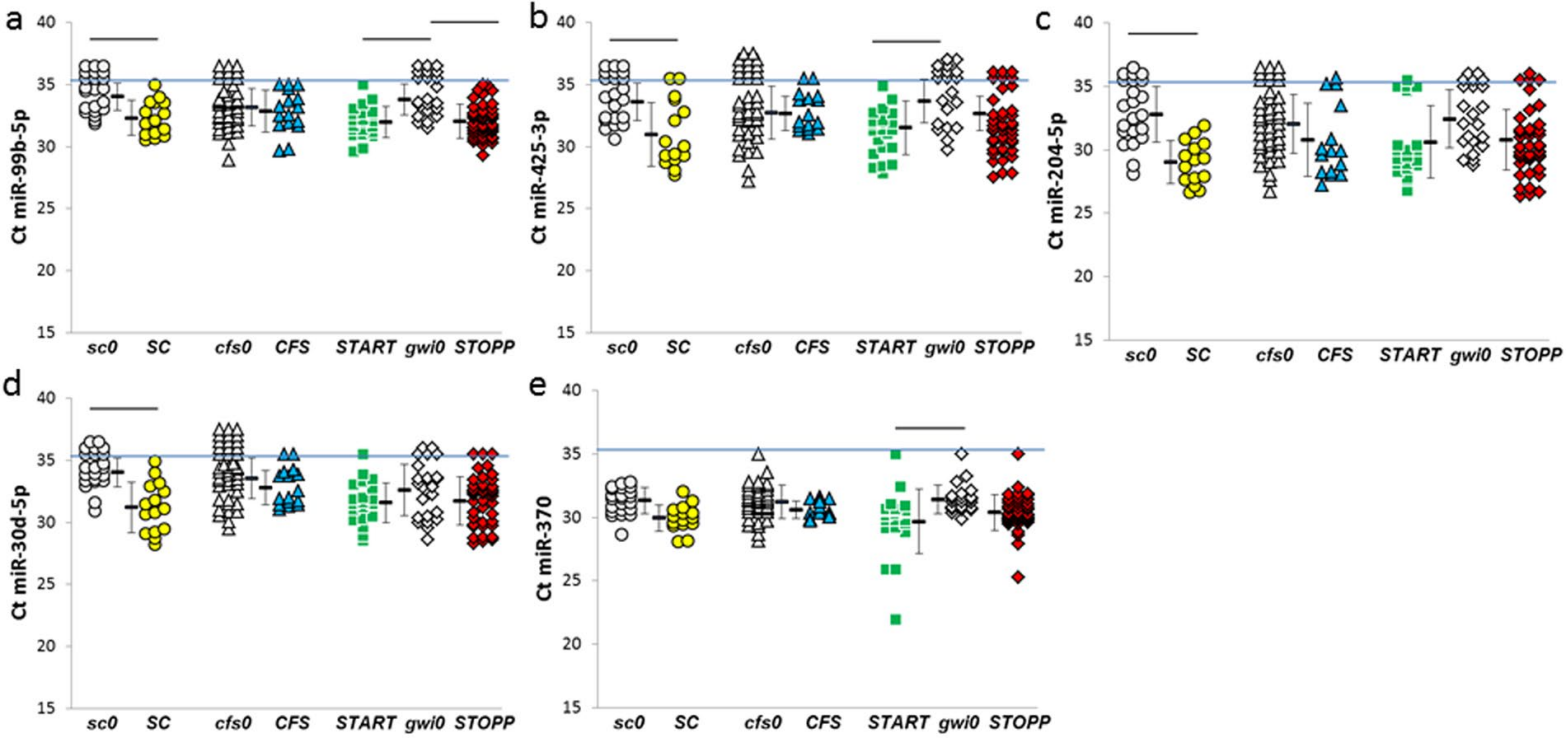
miRNAs that were significantly elevated in post-exercise compared to appropriate nonexercise control groups. Significant differences between groups were indicated by the bars at the top of the graphs for (**a**) miR-99b-5p, (**b**) miR-425-3p, (**c**) miR-30d-5p, (**d**) miR-204-5p, and (**e**) miR-370 (HSD ≤ 0.05, FDR ≤ 0.10, detected with Ct ≤ 35 in more than two thirds of one group per pair). The horizontal blue line indicated Ct = 35. Mean ± SD.

**Figure 4:**
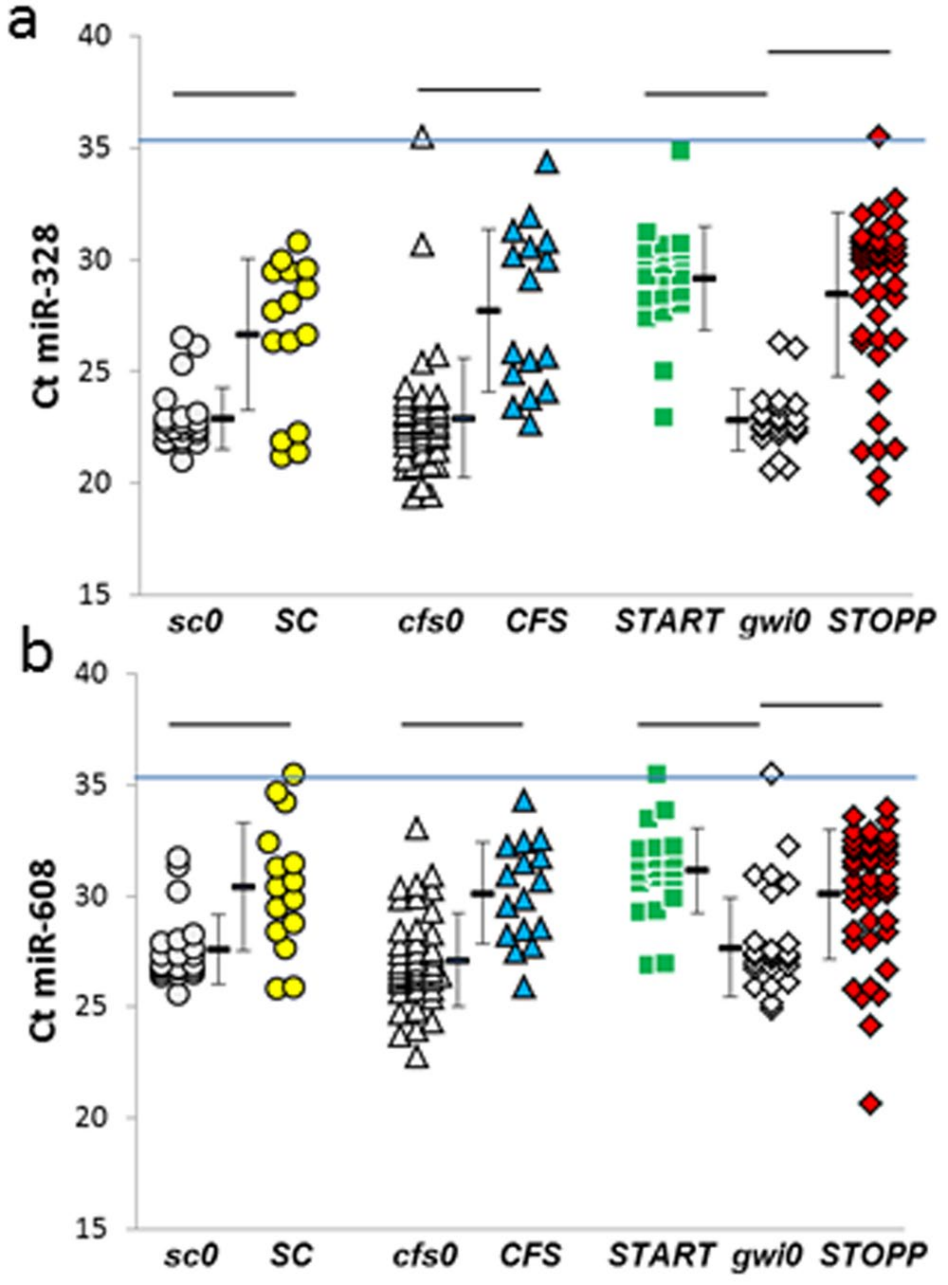
Decreased (**a**) miR-328 and (**b**) miR-608 levels in ***SC***, ***CFS***, ***START*** and ***STOPP*** groups after exercise.

**Figure 5:**
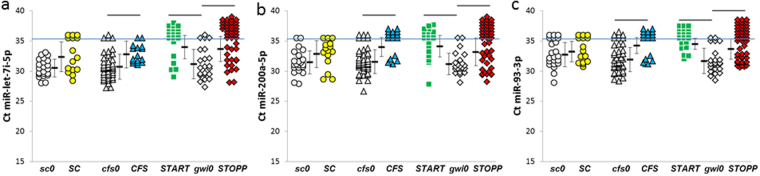
miRNAs reduced by exercise. GWI phenotypes (***START*** and ***STOPP***) and ***CFS*** all had reductions in (**a**) miR-let-7i-5p, (**b**) miR-200a-5p and (**c**) miR-93-3p. Sedentary controls had no changes.

**Figure 6:**
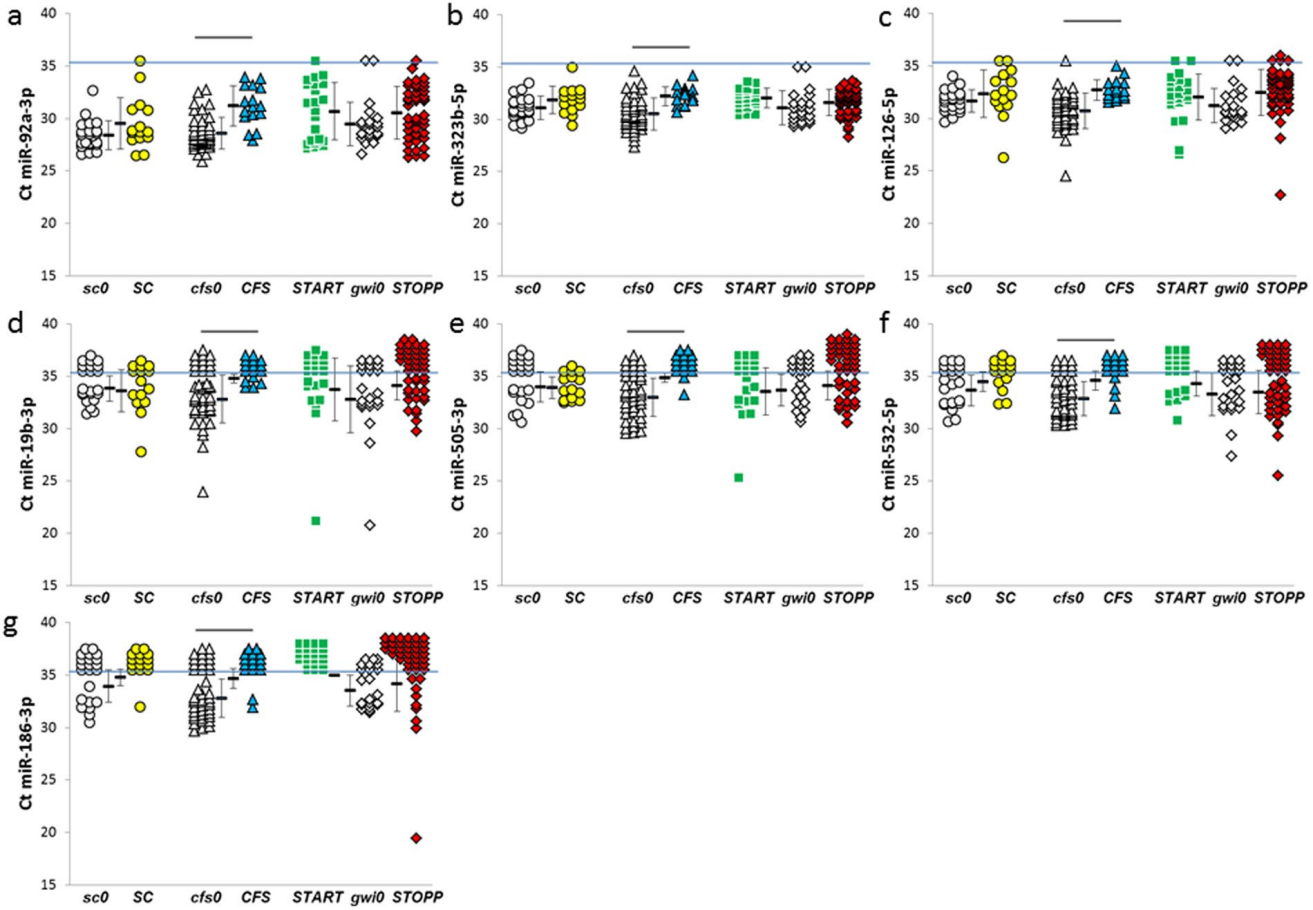
Decreased miRNAs after exercise in ***CFS*** group. ***CFS*** had significant reductions in -(**a**) 92a-3p, (**b**) miR-323b-5p, (**c**) miR-126-5p, (**d**) miR-19b-3p, (**e**) miR-505-3p, (**f**) miR-532-5p, and (**g**) miR-186-3p, compared to its nonexercise ***cfs0*** comparison group (bars above the groups).

