# Supplementary material for: Exercise – induced changes in cerebrospinal fluid miRNAs in Gulf War Illness, Chronic Fatigue Syndrome and sedentary control subjects
Source: Sci Rep. 2017 Nov 10;7:15338. doi: 10.1038/s41598-017-15383-9 (PMC5681566; doi:10.1038/s41598-017-15383-9)
Supplement: Supplementary file 2 — SOM Tables 2,4,5,6 [file 41598_2017_15383_MOESM2_ESM.doc]

SREP-17-04692-C

"***Exercise – induced changes in cerebrospinal fluid miRNAs in Gulf War Illness, Chronic Fatigue***

***Syndrome and sedentary control subjects***"

James N. Baraniuk 1*¶

Narayan Shivapurkar 1¶

1 Division of Rheumatology, Immunology and Allergy, Department of Medicine, Georgetown University, Washington, District of Columbia, United States of America

* Corresponding author

E-mail baraniuj@georgetown.edu

Version 2 Resubmitted July 18, 2017 SREP-17-04692-A

Version 3 Resubmitted August 15, 2017 SREP-17-04692-B

Version 4 Resubmitted October 7, 2017 SREP-17-04692-C

Supplementary Table S1. QPCR raw Ct for all subjects and all 380 miRNAs and 4 internal standards. Attached.

Supplementary Table S2. Abundance of miRNAs detected in cerebrospinal fluid. miRNAs were considered to be candidate biomarkers if they were detected with Ct≤35 in at least two thirds of one group. The numbers of subjects per group (N) and thresholds for two thirds per group were reported. miRNAs detected in less than two thirds of a group were highlighted in grey.

|  | Number of subjects per group with undetectable levels of mRNAs (Ct>35) | | | | | | |
| --- | --- | --- | --- | --- | --- | --- | --- |
|  | Nonexercise groups | | | Post-exercise groups | | | |
|  | sc0 | cfs0 | gwi0 | SC | CFS | START | STOPP |
| N | 22 | 43 | 22 | 15 | 16 | 22 | 42 |
| Threshold | 8 | 15 | 8 | 6 | 6 | 8 | 9 |
| Normalizers |  |  |  |  |  |  |  |
| miR-489 | 0 | 0 | 0 | 0 | 0 | 0 | 0 |
| miR-490-3p | 0 | 0 | 0 | 0 | 0 | 0 | 0 |
| miR-124-3p | 0 | 0 | 0 | 0 | 0 | 0 | 0 |
| miR-433 | 0 | 0 | 1 | 0 | 0 | 1 | 0 |
| miR-127-3p | 0 | 0 | 1 | 0 | 0 | 1 | 0 |
| miR-183-3p | 0 | 0 | 2 | 0 | 0 | 0 | 0 |
|  |  |  |  |  |  |  |  |
| miR-375 | 0 | 0 | 2 | 0 | 0 | 0 | 0 |
| miR-328 | 0 | 1 | 0 | 0 | 0 | 0 | 1 |
| miR-411-5p | 0 | 0 | 1 | 0 | 0 | 1 | 0 |
| miR-29a-3p | 0 | 1 | 1 | 1 | 0 | 0 | 0 |
| miR-323b-5p | 0 | 0 | 2 | 1 | 0 | 0 | 0 |
| miR-431-5p | 0 | 0 | 2 | 0 | 0 | 1 | 0 |
| miR-608 | 0 | 0 | 1 | 1 | 0 | 1 | 0 |
| miR-370 | 0 | 1 | 1 | 0 | 0 | 1 | 1 |
| miR-382-5p | 0 | 0 | 2 | 2 | 0 | 0 | 0 |
| miR-661 | 1 | 1 | 1 | 1 | 0 | 0 | 0 |
| miR-92a-3p | 0 | 0 | 1 | 1 | 0 | 1 | 1 |
| miR-187-5p | 0 | 2 | 1 | 0 | 0 | 1 | 1 |
| miR-342-3p | 0 | 1 | 0 | 0 | 0 | 2 | 2 |
| miR-484 | 0 | 1 | 2 | 1 | 0 | 1 | 0 |
| miR-320b | 0 | 4 | 1 | 0 | 2 | 0 | 0 |
| miR-181c-3p | 1 | 3 | 2 | 0 | 0 | 1 | 1 |
| miR-205-3p | 1 | 1 | 1 | 0 | 1 | 1 | 3 |
| miR-720 | 2 | 2 | 3 | 0 | 0 | 1 | 1 |
| miR-126-5p | 0 | 1 | 2 | 2 | 1 | 2 | 4 |
| miR-765 | 1 | 1 | 3 | 4 | 2 | 2 | 1 |
| miR-92b-3p | 0 | 2 | 1 | 1 | 1 | 3 | 9 |
| let-7b-5p | 3 | 5 | 4 | 0 | 3 | 4 | 5 |
| miR-25-5p | 2 | 7 | 5 | 1 | 1 | 3 | 5 |
| miR-421 | 3 | 2 | 6 | 1 | 0 | 6 | 6 |
| miR-210 | 7 | 8 | 4 | 2 | 0 | 3 | 3 |
| miR-320a | 4 | 4 | 3 | 2 | 4 | 2 | 9 |
| miR-324-3p | 5 | 7 | 6 | 1 | 1 | 3 | 6 |
| miR-423-3p | 4 | 14 | 4 | 3 | 3 | 4 | 4 |
| let-7c | 8 | 8 | 5 | 0 | 3 | 5 | 9 |
| miR-202-3p | 12 | 8 | 5 | 1 | 5 | 5 | 12 |
| miR-331-5p | 6 | 15 | 6 | 5 | 2 | 5 | 10 |
| miR-204-5p | 7 | 9 | 8 | 0 | 4 | 4 | 4 |
| let-7a-5p | 7 | 13 | 8 | 0 | 4 | 3 | 8 |
| miR-34c-3p | 0 | 2 | 1 | 2 | 3 | 7 | 16 |
| miR-4258 | 7 | 17 | 9 | 2 | 3 | 3 | 11 |
| miR-99b-5p | 9 | 9 | 9 | 1 | 3 | 1 | 1 |
| let-7e-5p | 9 | 13 | 11 | 0 | 3 | 5 | 10 |
| miR-425-3p | 9 | 14 | 12 | 2 | 2 | 0 | 6 |
| miR-30d-5p | 8 | 15 | 5 | 0 | 2 | 1 | 2 |
| miR-30a-5p | 8 | 21 | 10 | 3 | 3 | 1 | 4 |
| miR-30e-5p | 10 | 24 | 11 | 1 | 3 | 2 | 6 |
| miR-1180 | 13 | 18 | 6 | 2 | 4 | 6 | 19 |
| miR-200a-5p | 2 | 6 | 2 | 3 | 11 | 15 | 22 |
| miR-191-3p | 8 | 9 | 5 | 2 | 5 | 11 | 20 |
| miR-1224-5p | 12 | 13 | 6 | 4 | 8 | 10 | 12 |
| miR-22-3p | 12 | 10 | 9 | 3 | 2 | 15 | 8 |
| miR-21-5p | 8 | 12 | 10 | 0 | 4 | 7 | 16 |
| miR-195-3p | 13 | 12 | 14 | 4 | 6 | 8 | 21 |
| miR-505-3p | 13 | 13 | 11 | 4 | 14 | 12 | 26 |
| miR-486-5p | 11 | 15 | 9 | 2 | 6 | 6 | 14 |
| let-7f-5p | 12 | 18 | 14 | 5 | 7 | 8 | 21 |
| miR-1207-5p | 15 | 22 | 13 | 5 | 11 | 4 | 20 |
| miR-23a-3p | 14 | 23 | 14 | 3 | 7 | 6 | 25 |
| miR-33b-5p | 8 | 16 | 8 | 2 | 6 | 9 | 15 |
| miR-326 | 13 | 16 | 14 | 4 | 7 | 10 | 21 |
| miR-125b-5p | 13 | 18 | 11 | 5 | 6 | 9 | 19 |
| miR-26b-5p | 14 | 26 | 15 | 5 | 7 | 11 | 23 |
| miR-346 | 14 | 21 | 16 | 5 | 13 | 12 | 24 |
| miR-30c-5p | 16 | 23 | 11 | 4 | 10 | 12 | 22 |
| miR-26a-5p | 17 | 27 | 15 | 4 | 7 | 9 | 20 |
| miR-639 | 19 | 26 | 10 | 5 | 13 | 15 | 26 |
| miR-145-5p | 20 | 26 | 15 | 5 | 10 | 12 | 28 |
| miR-423-5p | 20 | 32 | 21 | 5 | 10 | 11 | 25 |
| let-7i-5p | 0 | 4 | 4 | 6 | 6 | 16 | 26 |
| miR-93-3p | 6 | 7 | 5 | 6 | 11 | 15 | 24 |
| miR-31-5p | 6 | 18 | 6 | 6 | 7 | 9 | 18 |
| miR-708-5p | 11 | 14 | 5 | 9 | 8 | 10 | 22 |
| miR-770-5p | 13 | 9 | 9 | 8 | 7 | 11 | 21 |
| miR-532-5p | 9 | 9 | 9 | 10 | 12 | 15 | 21 |
| miR-101-5p | 8 | 12 | 9 | 8 | 10 | 12 | 29 |
| miR-19b-3p | 10 | 13 | 9 | 7 | 11 | 13 | 24 |
| miR-196a-5p | 12 | 13 | 10 | 9 | 9 | 17 | 27 |
| miR-218-1-3p | 11 | 14 | 8 | 8 | 10 | 12 | 26 |
| miR-186-3p | 14 | 14 | 9 | 14 | 14 | 22 | 33 |
| miR-455-5p | 8 | 18 | 6 | 11 | 10 | 14 | 31 |
| miR-193b-5p | 9 | 19 | 12 | 6 | 5 | 5 | 15 |
| miR-339-3p | 12 | 15 | 11 | 7 | 7 | 8 | 18 |
| miR-205-5p | 14 | 24 | 11 | 6 | 6 | 8 | 16 |
| miR-125a-5p | 14 | 24 | 10 | 6 | 8 | 6 | 21 |
| miR-335-5p | 16 | 26 | 10 | 7 | 10 | 8 | 19 |
| miR-9-3p | 19 | 29 | 18 | 8 | 12 | 7 | 33 |
| miR-129-2-3p | 17 | 38 | 15 | 11 | 10 | 7 | 18 |

Supplementary Table S3. Summary calculations for N0 normalizer. The raw data (Supplementary Table 1) had Ct=35 imputed for every Ct>35. The mean ΔCt and standard deviation was calculated for every group and miRNA. ΔΔCt, the difference between each pair of groups, was calculated. Significance was determined between groups for each miRNA by ANOVA (p≤0.05) followed by Tukey’s Honest Significant Different (HSD, p≤0.05). False Discovery Rate (FDR) corrected for multiple comparisons FDR (p≤0.10). Attached.

Supplementary Table S4. miRNAs elevated after exercise. ΔΔCt values (mean±SD) were given for each miRNA that was significantly elevated after exercise compared to its appropriate nonexercise group. ΔΔCt for each normalizer (N0, N2, N3, N6) indicated high levels of agreement. Three of the 4 normalizers had to be significant for each miRNA.

| miRNA | Normalizer | SC>sc0 | CFS>cfs0 | START>gwi0 | STOPP>gwi0 |
| --- | --- | --- | --- | --- | --- |
| miR-22-3p | N0 | 6.03±5.34* | 2.22±4.45† | 1.72±4.57 | 3.88±5.54 |
|  | N2 | 5.91±5.34* | 2.06±4.51† | 1.87±4.62† | 3.48±5.56† |
|  | N3 | 5.91±5.37* | 2.01±4.49 | 1.80±4.63 | 3.54±5.53 |
|  | N6 | 5.61±5.20* | 2.08±4.41 | 1.94±4.46 | 3.44±5.38† |
| miR-30d-5p | N0 | 2.82±2.08* | 0.76±1.61 | 1.02±1.91 | 0.88±2.03 |
|  | N2 | 2.70±2.10* | 0.59±1.63 | 0.87±2.03 | 0.47±1.93 |
|  | N3 | 2.70±2.07* | 0.54±1.63 | 0.94±2.00 | 0.54±1.91 |
|  | N6 | 2.40±1.88* | 0.62±1.52 | 0.80±1.74 | 0.44±1.74 |
| miR-204-5p | N0 | 3.77±2.72* | 1.27±2.54 | 1.82±2.72† | 2.02±2.54* |
|  | N2 | 3.65±2.97* | 1.10±2.70 | 1.67±2.89 | 1.62±2.74 |
|  | N3 | 3.65±2.95* | 1.05±2.72 | 1.74±2.94† | 1.68±2.74 |
|  | N6 | 3.35±2.73* | 1.13±2.64 | 1.61±2.75 | 1.59±2.58 |
| miR-425-3p | N0 | 2.64±2.36* | 0.07±1.96 | 2.54±2.21* | 2.15±2.27* |
|  | N2 | 2.51±2.48 | 0.09±2.21 | 2.40±2.34* | 1.75±2.32 |
|  | N3 | 2.52±2.43* | 0.14±2.12 | 2.46±2.41* | 1.82±2.31 |
|  | N6 | 2.21±2.19* | 0.07±2.02 | 2.33±2.32* | 1.72±2.08* |
| miR-99b-5p | N0 | 1.71±1.49* | 0.30±1.55 | 1.82±1.53* | 1.74±1.56* |
|  | N2 | 1.59±1.31* | 0.14±1.58 | 1.67±1.53* | 1.34±1.53* |
|  | N3 | 1.59±1.31* | 0.09±1.62 | 1.74±1.53* | 1.40±1.53* |
|  | N6 | 1.29±1.15* | 0.16±1.40 | 1.60±1.33* | 1.31±1.33* |
| miR-370 | N0 | 1.37±1.22 | 0.65±1.20 | 1.74±2.12* | 1.03±1.40 |
|  | N2 | 1.25±1.15† | 0.49±1.23 | 1.59±1.87* | 0.63±1.31 |
|  | N3 | 1.25±1.02† | 0.44±1.08 | 1.66±1.90* | 0.70±1.31 |
|  | N6 | 0.94±0.98 | 0.51±1.14 | 1.52±1.98 | 0.60±1.17 |

* Significantly different by ANOVA, Tukey HSD (p≤0.05) and FDR (p≤0.10), and detected in more than two thirds of subjects in at least one group per pair. † Not significant because of Tukey HSD>0.05. All others were not significant by FDR>0.10.

Supplementary Table S5. miRNAs diminished after exercise. ΔΔCt values (mean±SD) were given for each miRNA that was significantly diminished after exercise compared to the appropriate nonexercise group. ΔΔCt was given for each normalizer (N0, N2, N3, N6) to show the level of agreement. Three of the 4 normalizers had to be significant for each miRNA.

| miRNA | Normalizer | sc0>SC | cfs0>CFS | gwi0>START | gwi0>STOPP |
| --- | --- | --- | --- | --- | --- |
| miR-608 | N0 | 2.81±2.58* | 3.01±2.51* | 3.47±2.71* | 2.41±2.92* |
|  | N2 | 2.94±2.71* | 3.17±2.56* | 3.62±2.72* | 2.81±2.91* |
|  | N3 | 2.94±2.75* | 3.22±2.58* | 3.55±2.65* | 2.74±2.87* |
|  | N6 | 3.24±2.85* | 3.15±2.53* | 3.69±2.68* | 2.84±2.85* |
| miR-328 | N0 | 3.75±3.01* | 4.84±3.65* | 6.36±3.73* | 5.64±4.09* |
|  | N2 | 3.88±3.18* | 5.01±3.76* | 6.51±3.82* | 6.04±4.11* |
|  | N3 | 3.88±3.19* | 5.06±3.78* | 6.44±3.84* | 5.98±4.11* |
|  | N6 | 4.18±3.25* | 4.98±3.82* | 6.58±3.93* | 6.07±4.10* |
| let-7i-5p | N0 | 1.86±2.13 | 2.05±2.29* | 2.84±2.61* | 2.50±2.49* |
|  | N2 | 1.98±2.25† | 2.22±2.29* | 2.99±2.66* | 2.90±2.60* |
|  | N3 | 1.98±2.27† | 2.27±2.34* | 2.92±2.65* | 2.84±2.61* |
|  | N6 | 2.28±2.29 | 2.19±2.28* | 3.06±2.66* | 2.93±2.53* |
| miR-93-3p | N0 | 0.53±1.88 | 2.32±2.10* | 2.82±2.14* | 1.99±2.04* |
|  | N2 | 0.65±2.07 | 2.48±2.27* | 2.97±2.28* | 2.39±2.18* |
|  | N3 | 0.65±2.10 | 2.53±2.29* | 2.90±2.21* | 2.33±2.16* |
|  | N6 | 0.96±2.03 | 2.46±2.11* | 3.04±2.20* | 2.42±2.10* |
| miR-200a-5p | N0 | 1.39±2.13 | 2.42±2.17* | 2.93±2.30* | 2.01±2.33* |
|  | N2 | 1.52±2.47 | 2.58±2.27* | 3.08±2.40* | 2.41±2.47* |
|  | N3 | 1.52±2.40 | 2.63±2.25* | 3.01±2.33* | 2.34±2.42* |
|  | N6 | 1.82±2.40 | 2.55±2.16* | 3.15±2.30* | 2.44±2.38* |
| miR-92a-3p | N0 | 1.14±1.93 | 2.60±1.99* | 1.21±2.47 | 1.08±2.41 |
|  | N2 | 1.27±2.05 | 2.76±2.20* | 1.36±2.61 | 1.48±2.43 |
|  | N3 | 1.27±2.08 | 2.81±2.18* | 1.29±2.72 | 1.42±2.55 |
|  | N6 | 1.57±2.01 | 2.74±2.16* | 1.43±2.75 | 1.51±2.44 |
| miR-126-5p | N0 | 0.67±1.65 | 1.98±1.76* | 0.84±1.97 | 1.24±2.09 |
|  | N2 | 0.80±1.86 | 2.14±2.02* | 0.99±1.82 | 1.64±2.13 |
|  | N3 | 0.80±1.76 | 2.19±1.93* | 0.92±1.91 | 1.57±2.11 |
|  | N6 | 1.10±1.71 | 2.12±1.81* | 1.06±1.84 | 1.67±2.09 |
| miR-19b-3p | N0 | 0.24±1.56 | 1.96±2.15* | 0.96±3.10 | 1.36±2.25§ |
|  | N2 | 0.12±1.83 | 2.12±2.29* | 1.11±3.30 | 1.76±2.48§ |
|  | N3 | 0.12±1.83 | 2.17±2.22* | 1.04±3.28 | 1.69±2.43§ |
|  | N6 | 0.18±1.75 | 2.10±2.11* | 1.18±3.31 | 1.79±2.39§ |
| miR-505-3p | N0 | 0.09±1.29 | 1.89±1.79* | 0.15±1.91 | 0.27±1.44 |
|  | N2 | 0.03±1.46 | 2.06±2.02* | 0±2.01 | 0.67±1.65 |
|  | N3 | 0.03±1.48 | 2.11±1.92* | 0.07±2.11 | 0.61±1.66 |
|  | N6 | 0.33±1.36 | 2.03±1.82* | 0.06±2.16 | 0.70±1.52 |
| miR-186-3p | N0 | 0.85±1.34 | 1.85±1.81* | 1.46±1.26§ | 0.62±2.30 |
|  | N2 | 0.97±1.70 | 2.01±1.98* | 1.61±1.36§ | 1.02±2.22 |
|  | N3 | 0.97±1.70 | 2.06±2.03* | 1.54±1.35§ | 0.96±2.29 |
|  | N6 | 1.28±1.61 | 1.98±1.89* | 1.68±1.37§ | 1.06±2.33 |
| miR-532-5p | N0 | 0.86±1.35 | 1.72±1.66* | 0.99±1.74 | 0.19±2.05 |
|  | N2 | 0.99±1.62 | 1.88±1.59* | 1.14±1.94 | 0.59±2.24 |
|  | N3 | 0.98±1.58 | 1.93±1.60* | 1.07±1.92 | 0.52±2.22 |
|  | N6 | 1.29±1.51 | 1.86±1.53* | 1.20±2.10 | 0.62±2.24 |
| miR-323b-5p | N0 | 0.76±1.23 | 1.68±1.57* | 0.93±1.38 | 0.51±1.41 |
|  | N2 | 0.88±1.43 | 1.84±1.64* | 1.07±1.42 | 0.91±1.39 |
|  | N3 | 0.88±1.49 | 1.89±1.67* | 1.01±1.45 | 0.84±1.40 |
|  | N6 | 1.18±1.39 | 1.82±1.53* | 1.14±1.31* | 0.94±1.22 |

* Significantly different by ANOVA, Tukey HSD (p≤0.05) and FDR (p≤0.10), and detected in more than two thirds of subjects in at least one group per pair. † Not significant because of Tukey HSD>0.05. § Not significant because not detectable with Ct≤35 in either group per pair. All others were not significant by FDR>0.10.

Supplementary Table S6. miRNAs that were significantly different between post-exercise groups. ΔΔCt values (mean±SD) for miR-22-3p and miR-9-3p were consistent for each normalizer. Large standard deviations for miR-22-3p were due to the wide spread of the data. The low magnitude differences for miR-9-3p were because this miRNA was only detectable in START.

| miRNA | Normalizer | SC>START | STOPP>START | START>STOPP |
| --- | --- | --- | --- | --- |
| miR-22-3p | N0 | 5.86±5.50* | 5.60±5.71* |  |
|  | N2 | 5.78±5.44* | 5.35±5.65* |  |
|  | N3 | 5.71±5.45* | 5.34±5.61* |  |
|  | N6 | 5.70±5.42* | 5.38±5.62* |  |
| miR-9-3p | N0 | 0.77±1.72 |  | 1.56±1.43* |
|  | N2 | 0.85±1.87 |  | 1.81±1.70* |
|  | N3 | 0.91±1.86 |  | 1.82±1.65* |
|  | N6 | 0.93±1.85 |  | 1.78±1.60* |

* Significantly different by ANOVA, Tukey HSD (p≤0.05) and FDR (p≤0.10), and detected in more than two thirds of subjects in at least one group per pair.
